# International Round Robin Test of Thermoelectric Generator Modules

**DOI:** 10.3390/ma15051627

**Published:** 2022-02-22

**Authors:** Pawel Ziolkowski, Przemyslaw Blaschkewitz, Byungki Ryu, SuDong Park, Eckhard Müller

**Affiliations:** 1 German Aerospace Center (DLR)—Institute of Materials Research, Linder Hoehe, D-51147 Cologne, Germany; przemyslaw.blaschkewitz@dlr.de (P.B.); eckhard.mueller@dlr.de (E.M.); 2 Energy Conversion Research Center, Electrical Materials Research Division, Korea Electrotechnology Research Institute (KERI), Changwon 51543, Korea; byungkiryu@keri.re.kr (B.R.); john@keri.re.kr (S.P.); 3 Institute of Inorganic and Analytical Chemistry, Justus Liebig University Gießen, Heinrich-Buff-Ring 17, D-35392 Gießen, Germany

**Keywords:** thermoelectric generator module, metrology, reference sample, standardization, uncertainty, round robin

## Abstract

The status of metrology for the characterization of thermoelectric generator modules (TEM) is investigated in this work by an international round robin (RR) test including twelve laboratories from nine countries on three continents. Measurements have been performed with three samples of a Bi_2_Te_3_-based commercial TEM type, which has prevailed over three competing types during previous tests on the short- and long-term stability. A comparison of temperature-dependent results is provided up to 200 °C hot side temperature for the maximum power output *P*_max_, the incident heat flow ${\dot{Q}}_{\mathrm{In}}$ (at maximum efficiency conditions), and the maximum efficiency *η*_max_. Data evaluation from all RR participants reveals maximum standard deviations for these measurands of 27.2% (*P*_max_), 59.2% (${\dot{Q}}_{\mathrm{In}}$), and 25.9% (*η*_max_). A comparison between RR data sets and reference data from manufacturer specifications shows high deviations of up to 46%, too. These deviations reflect the absence of measurement guidelines and reference samples and confirm the need for improvements in the standardization of TEM metrology. Accordingly, the results of the RR are presented against the background of our own investigations on the uncertainty budgets for the determination of the abovementioned TEM properties using inhouse-developed characterization facilities, which comprise reference and absolute measurement techniques for the determination of heat flow.

## 1. Introduction

**Figure 1:**
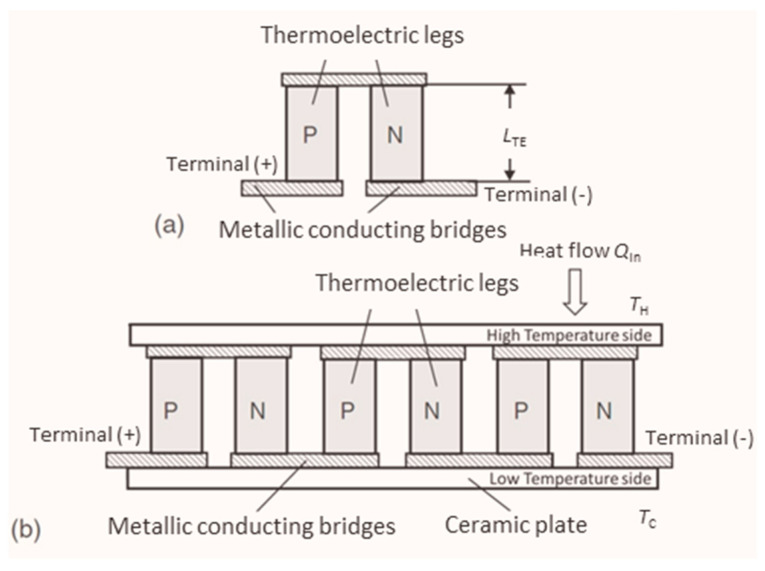
N- and p-type thermoelectric legs form a thermocouple (**a**). Multiple thermocouples can be connected electrically in series within a TEM (**b**). The TEM is operated between a heat source at the hot side temperature *T*_H_ and a heat sink with a cold side temperature *T*_C_. The heat flow ${\dot{Q}}_{\mathrm{In}}$ is partially converted into electric energy.

Thermoelectric generator (TEG) systems are suited to supply electric energy by the direct conversion of waste heat from mobile applications [1,2,3,4] and stationary processes [5,6,7]. TEGs consist of one or multiple thermoelectric generator modules (TEM), which operate within heat transmission paths between heat reservoirs. Every TEM includes an electric series connection of a variable number of p- and n-type thermoelectric (TE) legs (Figure 1). These connections are formed by metallic bridges, which shall have low electric contact resistances for low parasitic internal power dissipation. Secondly, a high heat transfer coefficient at interfaces is needed in order to maximize the effective temperature difference along the TE legs, which is the driving force for the generation of a thermovoltage *V*_0_ due to the Seebeck effect [8]. Assuming constant material properties, the thermovoltage *V*_0_ of a TEM can be expressed as

(1) $$V_{0}=N\cdot \left(S_{p}-S_{n}\right)\cdot \left(T_{H}-T_{C}\right)\;=S\cdot ∆T$$

Here, *N* is the number of thermocouples in a TEM, while *S*_p_, *S*_n_, *T*_H_, and *T*_C_ denote the Seebeck coefficients of the p-/n-type TE legs and the hot and cold side temperature, respectively. The effective Seebeck coefficient of the TEM is defined as *S* = *N*·(*S*_p_ − *S*_n_), with the temperature difference Δ*T* = *T*_H_ − *T*_C_. Δ*T* is typically measured in the vicinity of the sample within adjacent components for heat exchange, which are installed inside the measuring section in a thermal serial connection to the TEM under test. Such measurements consequently involve temperature drops at the interfaces of the TEM coupling faces. Thus, the thermal contact resistances of the module under test become an inherent part of the derived TEM properties and should be reproduced according to the installation conditions of the later application to the highest possible degree. The open-loop voltage *V*_0_ can be tapped at the terminals of the TEM at zero electric current (*I* = 0). Under current flow (*I* ≠ 0), the terminal voltage *V* of the module is reduced by the ohmic voltage drop owing to its electric resistance *R*.

(2) $$V=V_{0}-I\cdot R$$

The power output *P* of a TEM equals the product of the terminal voltage and the electric current.

(3) $$P=V\cdot I=S\cdot ∆T\cdot I-I^{2}\cdot R\;$$

The maximum power output *P*_max_ is delivered by a TEM, if the electric load resistance *R*_L_ matches the internal resistance *R* of the TEM, which can be expressed by a load ratio m = *R*_L_/*R* = 1 [9].

(4) $$P=\frac{V_{0}^{2}}{R}\cdot \frac{m}{{\left(m+1\right)}^{2}}\;\overset{m=1}{\to }\;\;P_{max}=\frac{V_{0}^{2}}{4R}$$

The efficiency *η*_TEM_ of a TEM is defined by dividing the electric power output *P* by the incident heat flow ${\dot{Q}}_{\mathrm{In}}$ at the hot side [10].

(5) $$\eta_{\mathrm{TEM}}=\frac{P}{{\dot{Q}}_{\mathrm{In}}}$$

As for any other heat engine, the Carnot efficiency *η*_C_ = (*T*_H_ − *T*_C_)/*T*_H_ represents the theoretically maximum achievable conversion efficiency *η*_max_. Loss mechanisms, which originate from material properties and electric and thermal contact resistances within the TEM, affect *η*_max_ additionally. These properties are combined within the module’s figure of merit *ZT*_TEM_ = *S*^2^/(*R*·*K*)·*T*_m_, with the mean temperature *T*_m_ = (*T*_H_ + *T*_C_)/2, *S* = *N*·(*S*_p_ − *S*_n_) being the effective Seebeck coefficient value, *K* the thermal conductance (at *I* = 0), and *R* the electric resistance of the module. All these module properties represent averaged values, which are calculated under consideration of the operation temperature range [10].

(6) $$\eta_{\max}=\frac{T_{H}-T_{C}}{T_{H}}\frac{\sqrt{1+ZT_{\mathrm{TEM}}}-1}{\sqrt{1+ZT_{\mathrm{TEM}}}+\frac{T_{C}}{T_{H}}}$$

Bi_2_Te_3_ represents an established TE material, which is commonly used for industrial TEM production [11,12,13,14,15,16,17]. Commercial TEMs with Bi_2_Te_3_ show maximum conversion efficiencies of 7.2% [18]. Many works investigated the improvement of the functional stability of Bi_2_Te_3_ using adapted temperature treatments [19,20,21] or by the altering of compositions [22,23,24]. Although measurements of the material figure of merit *zT* > 1 (*zT = S*_p−n_^2^⋅*σ*_p−n_/*κ*_p−n_, with *σ*_p−n_, *κ*_p−n_ being the electric and thermal conductivities of p- and n-type TE materials) have been demonstrated for Bi_2_Te_3_ up to hot side temperatures of 500 °C [25], the application of commercial Bi_2_Te_3_-TEMs remained below 300 °C [26], which is related to the limited stability of Bi_2_Te_3_ and the contacting of this material. Progress on contacting technologies and the improvement of high-temperature TE materials revealed mature TEM prototypes [27,28,29] and enabled the establishment of small batch production by industries [30,31,32,33]. Attractive TEM efficiencies between 8.9% [34] and 12% [35] have been demonstrated in recent years by laboratory prototypes with different high-temperature TE materials, effectively offering the possibility for future applications with temperature differences > 500 K. This progress increases the demand for reliable characterization techniques for TEM, which can be recognized as a prerequisite for the future industrialization of this technology and exploitation of markets by TE applications.

However, the lack of standardized characterization techniques, guidelines, and TEM reference samples still impedes the reliable specification of TEM properties. Development of high-precision characterization methods and their transformation into primary methods with specified uncertainty budgets and traceability of measurands is required. Several works with this focus have been accomplished in the field of TE material characterization, yielding uncertainty analyses for commercial or custom-made measurement systems for the determination of the Seebeck coefficient and the electric resistivity [36,37,38], as well as for *zT* measuring systems [39]. This has led to an increased standardization level of metrology for TE material properties, which is characterized to date by available reference samples [40,41], descriptive guidelines for the conduct of transport property measurements [42,43], and reports on comparative measurement campaigns [44,45,46,47].

Corresponding studies for TEM characterization techniques lag behind. Although recent works reported on starting initiatives for the development of TEM reference samples [48,49], their current lack still complicates the expression of uncertainty budgets. According to the “*Guide to the expression of uncertainty in measurement*” (GUM) [50], the definition of uncertainty budgets requires the determination of influencing parameters and sensitivity coefficients for every measurand, which raises high experimental effort in the absence of standardized reference samples. A statistically meaningful number of repetitive measurements is required for various TEM measurands and within a wide range of measurement boundary conditions. The level of complexity with regard to instrumentation, control of measurement conditions, and evaluation procedures for TEM metrology places high effort in such investigations, which might be one reason for which respective work has not been started on a broader front yet.

A brief presentation on reported approaches for TEM characterization and the level of confidence for the determination of TEM properties will be discussed in the beginning of Section 2 of this publication. However, available information from published works shows that only a few experimental studies have been accomplished. Herein, authors mainly demonstrate the functionality of developed devices and present measurement results without or with only basic discussion and consideration of measurement uncertainties. Uncertainties for measurements of central TEM properties, such as power output, heat flow, and efficiency, have neither been determined in accordance to the GUM nor reported before in the literature to the best of our knowledge.

In order to improve this situation, we provide further information on characterization facilities, which are operated at the German Aerospace Center (DLR). In addition to previous publications, which have quantified the uncertainty of heat flow measurement on a thermal reference material [51] and the uncertainty of module resistance measurements [52], we report on the uncertainty budget for the maximum power, heat flow, and efficiency measurement on a TEM in this work, in order to allow for a conclusive interpretation of observed deviations between round robin (RR) results. Besides discussion of the RR results, a brief summary of experiments for module sample selection will be given, in addition to further relevant information on the organization of comparative RR tests and their technical concept.

## 2. Materials and Methods

This section starts with a survey on reported measurement techniques for TEM characterization and a brief introduction to the inhouse-developed characterization facilities “TEGMA” and “A-TEGMA” (Absolute Thermoelectric Generator Measurement Apparatus). A detailed description of these apparatuses has been given in a previous work [51]. Here, we report on basic device operation and stabilization criteria only, which are relevant for the definition and quantification of uncertainty budgets for measurements of the power output, heat flow, and efficiency. Results of the uncertainty analyses are presented in Section 3. The analyses are based on experimental TEGMA results, which have been obtained on a comparative sample of the RR.

### 2.1. TEM Metrology Survey

Several approaches for TEM characterization are known from the literature. Techniques can be differentiated by transient and steady-state measurement approaches. Furthermore, they can be divided into methods that conclude on *η* from measurements of the heat flow and the electric power output according to Equation (5), or those that use Equation (6), effectively calculating the module efficiency on the basis of measured *ZT*_TEM_ values. Impedance spectroscopy [53] and the Harman method [54,55] represent transient methods, which measure *ZT*_TEM_ to conclude on *η*. However, steady-state characterization techniques with direct measurement of the power output and the heat flow are most common and yield more application-oriented predictions of TEM performance compared to the use of *ZT*_TEM_ and the subsequent calculation of *η* by Equation (6). One reason for this is the small temperature differences of a few K, which are typically applied for measurements of *ZT*_TEM_, whereas usual operation of TEMs implies several hundreds of K temperature difference. Generally, higher temperature differences have been applied for measurements of *ZT*_TEM_, too [56]. However, Equation (6) is based on a constant property model (CPM) using averaged values of temperature-dependent material properties [57]. Secondly, CPM neglects further relevant impacts on the operation and performance of TEMs, such as Thomson heat, asymmetric distribution of Joule heat, electric and thermal contact resistances, and the presence of parasitic heat bypasses by means of radiation or convection [58], which yields approximated values for *η* only. Armstrong et al. [59] studied the performance determination for 18 TE materials and showed an overestimated prediction of *η* (by an average factor 1.16) by CPM compared to a cumulative model, which was proposed earlier by Kim et al. [60] to account for the temperature dependence of material properties by means of the so-called “engineering figure of merit”. Even neglecting specific features of known analytic models for TEM efficiency prediction, any analytically based determination of TEM properties will suffer from elusive thermal and electrical coupling conditions and from uncertainties of input parameters, which are obtained by preceding TE property measurements on material samples.

Classic steady-state measurement techniques for TEM characterization mainly differ with regard to the applied approach for heat flow determination. The most widespread technique is the so-called reference principle [61,62,63], which uses reference blocks as heat flow meters (HFM). Technical details and uncertainty sources of this measurement principle have been described extensively, but only for thermal conductivity measurements on material samples by the standard test method ASTM-E1225 [64] and within scientific publications [65,66,67,68]. Analogous to thermal conductivity measurement, used reference blocks are made from materials with known thermal conductivity and placed thermally in series to the TEM under test. Temperature sensors inside the HFM give access to the average temperature gradient $\frac{\Delta T_{M}}{l_{M}}$, from which the heat flow ${\dot{Q}}_{Ref}$ can be deduced by application of the one-dimensional Fourier’s law.

(7) $${\dot{Q}}_{Ref}=-A_{M}\cdot \kappa_{M}\;\left(T\right)\cdot \nabla T_{M}=-A_{M}\cdot \kappa_{M}\;\cdot \frac{∆T_{M}}{l_{M}}\;$$

Here, $\kappa_{M}$, $A_{M}$, $∆T_{M}$, and $l_{M}$ denote the thermal conductivity, the cross-sectional area, the temperature difference, and the length of the HFM section, which is equipped with temperature sensors. The underlying uncertainty was analytically derived in our recent work [51], which considered uncertainty contributions from all input variables according to Equation (7). The study was conducted with an HFM made of the thermal reference material NPL 2I09 (Inconel 600) from the National Physical Laboratory (NPL) [69]. The combined uncertainty of heat flow determination $u\left(\dot{Q}\right)$ by the reference principle was experimentally determined in accordance to the GUM in a temperature range between 373 and 1023 K, yielding *u*(${\dot{Q}}_{Ref})\;$= 10–13% (*k* = 2). Here, *k* denotes the coverage or probability factor, which expresses the statistical uncertainty of a measured value and the corresponding confidence interval (*k* = 1/1.96 → 68%/95% confidence).

Previous works on the reference principle were mainly limited to descriptions of the employed setup and demonstration of their use for the characterization of lab prototype [61,62,70] or commercial [71,72,73] TEMs. Although measurement procedures and instrumentation for temperature-dependent heat flow measurements have been described in some of these works—for instance, König et al. [72] and Man et al. [73]—they finally did not present heat flow results at all, while Populoh et al. [70] gave only a single heat flow value for a particular temperature condition, without further discussion on its uncertainty. Hejtmanek et al. [71] admitted the heat flow to be the most difficult parameter to be measured and described temperature measurements at the center and the edges of the employed HFM, which was made from Cu and placed at the hot side of the tested TEM. Independently, the cold side heat flow was determined additionally from the specific heat and flow rate and the in- and outlet temperature of a coolant in the heat sink of the measurement setup. Despite thermal insulation inside the measuring chamber, Hejtmanek [71] reported heat losses of the used heater of approximately 25%. This finding was not discussed further but probably arose from differences in both methods for heat flow determination. Without further analyses on the accuracy of both methods, the authors stated a higher measured heat flow compared to manufacturer specifications of the tested TEM.

Beyond the reference principle, a measurement on a commercial TEM was demonstrated by Chien et al. [74]. In this work, one side of the sample was kept adiabatic. Measurements were conducted with a hot side heater in a temperature range between 21 and 155 °C. A maximum temperature difference of approximately 5 K was set by varying the electric current flow through the sample. The authors claimed errors of the average thermal conductivity of ±11%, while reporting ±2% and ±3% for the average Seebeck coefficient and resistivity, respectively. However, the authors equated measurement errors with the total scattering range of the respective measurands in dependence on the applied heater temperature and current flow setting. This is in conflict with the common definition of a measurement error, which is actually resulting from an evaluation of an underlying error propagation model. Such models are based on analytic functions for the determination of particular measurands and account for individual influencing parameters and their statistical distribution, which is well expressed by the corresponding standard deviation. Besides the fact that characterization results obtained on commercial TEMs have been compared to manufacturer specifications in rather few cases, the absence of error analyses or imprecise use of terminology reflect a lack of awareness, which can potentially lead to an incorrect perception of data confidence and inaccurate conclusions from scientific work.

Returning to the reference principle, measurements on inhouse-developed double leg configurations have been described by Groβ et al. [61] and Müller et al. [62], who compared their experimental results with analytically determined reference data on heat flow, thermal resistance, or efficiency, which were calculated on the basis of one-dimensional (1D) approximation models for TEM operation, with input parameters from transport property measurements on TE materials used in the couples. Although the distinct discussion and disclosure of quantitative deviations and experimental uncertainties was left also here, the authors pointed to several reasons for possible deviations, such as the quality of contacts or convective heat losses, and gave a visual indication of results’ agreement by figures, which reflected a decent accordance between experiments and analytic property predictions for the studied double leg configurations.

Takazawa et al. [63] quantified exemplarily a deviation between a single data point of measured heat flow and a reference value from calibration with a certified reference material for thermal conductivity. The authors used an oxygen-free highly conductive Cu (OFHC) HFM. This Cu-HFM was positioned at the cold side of the measuring section at approximately 300 K mean temperature, while a second HFM was placed thermally in series between the heater and the Cu-HFM. The HFM at the hot side with the same cross-section as the Cu-HFM was made of the certified standard reference material (SRM) for thermal conductivity, RM8420 (electrolytic iron), from the National Institute of Standards and Technology (NIST) [75]. Using this configuration, the thermal conductivity of the Cu-HFM was calibrated using the measured temperature gradient inside and heat flow data, which were determined from the gradient in the SRM-HFM and its certified values for thermal conductivity. However, the authors did not consider any heat flow deviation between both HFMs or the uncertainty of linear approximations of the temperature profiles inside both HFMs. Assessment of their reported results for this experiment [63] indicates a maximum scatter of the calibration data for the thermal conductivity of the Cu-HFM of approximately ±2.5 Wm^−1^·K^−1^ around 300 K. The temperature-dependent thermal conductivity of the Cu-HFM was finally defined by an unspecified line fit to the results of the calibration test. In a last step, Takazawa et al. characterized a dummy sample with known thermal conductivity in a following experiment. This dummy sample was made from quartz glass and was installed replacing the SRM-HFM. Without further comment on the reference value for the thermal conductivity of the dummy sample or citation of the underlying data source, a maximum deviation of <5% for the heat flow measured by the Cu-HFM was assumed. This deviation was attributed to a parasitic heat input into the calibrated cold side Cu-HFM by thermal radiation from the heater, which was possibly reflected by the environment of the experimental setup, effectively yielding an overestimation of the measured heat flow. The temperature dependence of heat flow data was not considered, nor were the uncertainty contributions and statistic distributions of measurands discussed.

Short et al. [76] tested a measurement system based on the reference principle using a reference sample made from fused quartz glass. This sample was connected thermally in series between a hot and a cold side reference block, yielding a two-fold heat flow determination. The hot side block was made from stainless-steel (SS-304), while OFHC was used at the cold side. Non-certified reference values for the thermal conductivity of both materials were taken from literature references [77,78], respectively, without further statement on their accuracy or applicability. Neither the resulting experimental uncertainty nor deviations between heat flow results and reference values have been quantified or discussed throughout the paper. Instead, graphically, the almost perfect linearity of *Q*_hot_ vs. *Q*_cold_ (from SS-304 and OFHC HFMs) was presented and good accordance of the measured thermal conductivity of the quartz sample to a reference by Sugawara [79] was concluded.

An absolute measurement technique is given by the guarded hot plate (GHP), which can be used for heat flow determination alternatively to the reference principle. A GHP transfers the heat flow determination into measurements of the voltage drop $V_{\mathrm{GHP}}$ and current flow $I_{\mathrm{GHP}}$ of a metering heater (MH) in order to determine its dissipated Joule heat.

(8) $${\dot{Q}}_{\mathrm{GHP}}=V_{\mathrm{GHP}}\cdot I_{\mathrm{GHP}}\;$$

Heat losses of the MH are minimized by actively controlled guard heaters. These guard heaters enclose the MH and yield desirably isothermal conditions in every direction except towards the sample under test. GHPs have been described as a standard test method for thermal conductivity measurements on monolithic material samples by the ISO-8302 [80] or ASTM-C177 [81]. The GHP method has been extensively studied over the last few decades with regard to its accuracy [82,83,84,85,86,87] and customized devices are available from companies for metrological equipment [88,89,90]. However, technical implementations of commercial products and the design described by ISO-8302 or ASTM-C177 differ significantly from a GHP for the characterization of TEMs. First, TEM samples come with a high variety of geometric designs, whereas commercial GHP devices require large sample geometries with lateral dimensions of several tens of cm. Secondly, TEMs have to be characterized under variable mechanical pressure and high temperature differences, which is neither offered by customized GHP products nor foreseen by respective normatives. Finally, TEMs have to be characterized electrically, too, which requires consideration of the Peltier effect [91] on the temperature distribution and heat flow along measuring sections in the first place. Instrumentation and adapted design for the accommodation of current leads and signal sensing are additionally needed for a meaningful test on a TEM but not offered by commercial GHP devices.

The few reports on the application of custom-built GHPs for TEM characterization can be split into concepts with passive thermal insulation and active temperature guarding of the MH. Montecucco et al. [92], Sandoz-Rosado et al. [93], and Zybala et al. [94] described GHP apparatuses with passive thermal insulation of the MH and proposed a correction of its measured power by estimates of heat losses to the environment.

Montecucco [92] calculated the conductive heat losses of the MH from the thermal resistance of the surrounding insulation and emerging temperature differences within the measurement system. The thermal resistance was calculated from the thermal conductivity and geometry of the insulation. Neither technical implementation of temperature sensors in the system nor observed temperature differences between the MH and its surrounding nor any results on calculated heat losses were reported. The measured efficiency and effective thermal conductivity of a commercial TEM were presented without any information on measurement uncertainties and without data comparison to product specifications.

Sandoz-Rosado [93] described a similar approach and gave analytic expressions for the parasitic heat bypass within a TEM due to radiation between the hot and the cold substrate surfaces. No information was given on technical implementations or the method for the determination of heat losses of the MH. The authors stated a temperature-dependent measurement of heat losses through the insulation of the MH and claimed a maximum uncertainty of these measurements of 3–5%, without indicating the magnitude of the heat loss itself. The heat flow measurement was validated by tests on two monolithic material samples made from Borofloat^®^ borosilicate and Macor^®^ glass ceramics. The deviation between the measured and specified thermal conductivity was reported as <4.2% (Borofloat^®^) and <6.5% (Macor^®^) [93], without a source of reference data cited. When characterizing a commercial TEM, consistency of data was shown visually, without discussion on statistic distributions of measurands or resulting uncertainties. The authors compared the measured power output, heat flow, and efficiency of the tested TEM with simulated values from a 1D-TEM model, without taking into account electric contact resistances, but considered thermal interface properties and TEM-internal heat leakage due to radiation between hot and cold substrates. Input data for the 1D model were partly given, e.g., for TEM properties but not for the assumed thermal conductivity of the measurement atmosphere or emissivity of ceramic plates. The authors gave a visual comparison between measured and simulated TEM efficiency and heat flow [93], involving considerable mismatch. Deviations between measured and simulated module efficiency and heat flow have not been quantified or discussed.

Zybala [94] described an apparatus comprising two independent methods for heat flow determination. Besides a GHP, which was used as a heat source, Zybala determined additionally the outgoing heat flow at the cold side of the TEM, similarly to Hejtmanek [71], from the specific heat, flow rate, and temperature difference between the coolant in- and outlet in the heat sink. The latter principle was reported earlier by Hu et al. [95], who stated the measurement of the flow rate as the dominant source of heat flow error. Hu indicated another systematic error contribution due to the intrinsic dissimilarity of the two temperature probes for coolant measurement but did not quantify or discuss the overall uncertainty or individual contributions. However, Zybala [94] reported thermal losses of the GHP to the environment in the range of 30% of the measured GHP power. This is in good accordance with the 25% heat loss of a passively insulated heater as reported by Hejtmanek [71]. Zybala [94] added the electric power output of the TEM and the cold side heat flow, which was measured at the heat sink, and subtracted this sum from the measured GHP power for an assessment of the GHP heat losses. No additional information on the uncertainty of heat flow determination by the GHP or at the heat sink was given. Discussion of TEM results obtained on a commercial type was limited to heat flow data from measurement at the heat sink. Zybala discussed variation of the thermal TEM coupling and resulting temperature deviations as possible reasons for the observed mismatch between the measured and specified power output and resistance of the tested TEM. Finally, the authors stated an overestimation of the maximum efficiency by the supplier data compared to their own experimental results, which was not traceable since the listed reference data in [94] did not include any information on the thermal resistance, heat flow, or efficiency of the TEM.

GHPs for heat flow measurement on TEMs involving actively controlled guard heaters for thermal shielding of the MH have been described by Anatychuk et al. [96], Kwon et al. [97], Rauscher et al. [98,99], and Ziolkowski et al. [51]. Anatychuk developed a GHP and tested the accuracy of the MH against a custom-made heat flux sensor, which was installed at the cold side of a tested TEM. This flux sensor was ingeniously fabricated as a thin plate-type HFM containing an internal thermopile, which was formed by a series connection of several thermocouples with areally distributed junctions between the hot and the cold coupling side of the plate. The thermopile effectively translates a cross-plane heat flow into a thermovoltage signal, which is proportional to the temperature difference over the plate-type HFM. The benefit of using a thermopile instead of single temperature sensors distributed in the axial direction of heat flow is given by the signal formation of the thermopile, which reflects a mean temperature difference averaged over both coupling planes of the HFM. Anatychuk presented the sensitivity of this heat flux sensor in a temperature range between 10 and 90 °C. As stated but not described, the uncertainty was experimentally investigated upon a ceramic dummy sample with a thermal resistance of 2 KW^−1^. The authors claimed a maximum scatter of heat flow data from the flux sensor <3%, which was likewise observed for measurements on commercial TEM types. Anatychuk described a measurement on an Altec-1060 generator module, which was tested at *T*_C_ = 30 °C and *T*_H_ = 300 °C, where the heat flux sensor measured a heat release at the cold side of the TEM of 140 W, while the GHP source produced 190 W of incident heat flow. Anatychuk attributed the observed heat flow difference to several heat losses of the MH to the environment, such as through the temperature-controlled casing (30 W), along supply leads (4 W), and by radiation of a so-called “hot heat-levelling plate” located between the GHP and the TEM sample (3 W). While 9 W was the converted electric power of the TEM, the missing 4 W from the energy balance was attributed to the heat flow measurement error of the heat flux sensor at the cold side of the configuration, and a maximum heat flow error <3% was claimed for the apparatus, which refers most likely to the plate-type heat flux sensor at the cold side but would be difficult to interpret as an uncertainty specification for the GHP in view of an estimate of 19% of heat loss. Although the authors mentioned an experimental quantification of the above-listed heat losses, no details or references were given. A follow-up publication of Anatychuk [100] revealed a data-sheet-like survey on the performance characteristics of commercial Altec modules, according to which the previously studied Altec-1060 TEM was specified with a lower maximum power output of 8 W, instead of 9 W as previously. This discrepancy and the reported heat loss level of the MH raise questions about the claimed heat flow error of the plate-type HFM, the efficiency of the active thermal guard, and the resulting heat flow uncertainty of the GHP, which effectively remained unspecified but was assessed non-traceably for a single temperature condition.

Kwon et al. [97] described a GHP apparatus with a rectangular-shaped MH and two actively controlled guard heaters. One guard was manufactured as a ring-shaped heater for the suppression of lateral heat losses, while a second guard was placed above the MH to minimize heat losses to the mechanical mounting. Kwon tested a commercial TEM of 40 mm × 40 mm in cross-section, while the size of the coupling area of the MH was specified as 30 mm × 30 mm. The protruding margin of the TEM outside the coupling area to the MH was thermally coupled to the ring-shaped guard heater and its gap towards the MH, which is in conflict with a configuration for thorough heat flow measurement by the MH and raises questions about the temperature homogeneity of the hot side of the TEM. Kwon finally did not present any heat flow results of the described GHP apparatus but showed a temperature-dependent measurement of the *ZT* value by means of the Harman principle, which does not require any heat flow measurement.

Rauscher et al. [99] reported a GHP apparatus with a single guard heater for the suppression of heat losses in an MH made from a heating wire, which was embedded within an AlN block. The guard heater was installed on top of the MH with a ceramic thermal insulation block in between. This insulation block was constructed with a flat cavity, which involved a pin-like support of the MH. This shape effectively ensured the thermal enclosure of the MH by the insulation block in every direction except towards the TEM under test, while simultaneously minimizing paths for a possible conductive heat exchange between the MH and the insulation block due to the local fixing of the MH. Instead of using single, localized temperature sensors for the temperature control of the guard heater, a thermopile configuration was used, which was installed in the thermal insulation block. This thermopile effectively formed thermocouple junctions on both sides of the insulation block and covered a large part of the opposite surfaces of the MH and the guard heater. This original solution is considered to provide a more meaningful temperature setpoint for the guard heater since the thermopile delivers a voltage signal, which is proportional to the mean temperature difference of both heater surfaces and less prone to uncertainties involved with the usage of a single sensor. Rauscher et al. tested the GHP apparatus on an SRM for the thermal conductivity, PR.41.08 (Nimonic 75 CrNi-Steel) from the NPL [101]. A reference block with a cross-section of 23.5 × 23.5 mm^2^ (matching the sample coupling area of the MH) and a height of 40 mm was manufactured from the reference material and equipped with two thermocouples positioned at its central axis at a distance of 30 mm in the direction of heat flow. The GHP test involved a power measurement of the MH and a heat flow measurement by the reference block, which was evaluated from the measured temperature difference inside the SRM and its known thermal conductivity. Rauscher estimated the uncertainty of the heat flow measurement by the reference block at ±5%, which was mainly caused by contributions of the specified thermal conductivity of the SRM (±3%), contributions of a potential displacement of temperature sensing points inside the reference block (±2.4%), and calibration uncertainties of the used thermocouples (±1.6%). Rauscher et al. specified a temperature-dependent deviation of the GHP-based heat flow compared to the outcome of the reference block of 2–3% in the temperature interval between 50 and 275 °C. The authors concluded that they could not decide from the acquired data which of the two heat flow measurements would provide more accuracy, since the deviation of the GHP was within the combined uncertainty of the reference value from the SRM.

A similar deviation of 2.25% between a GHP-based heat flow and a comparative value from an SRM-based reference block measurement was described by Ziolkowski et al. [51]. Here, the authors reported on reproducible findings from repetitive measurements, which revealed a temperature-dependent deviation of the GHP heat flow within the uncertainty limit of the SRM-based reference value. Ziolkowski et al. studied the thermal crosstalk between the MH and its guard heater system, which contained four individually controlled heater circuits. By varied detuning of the guard heater temperatures relatively to the MH temperature *T*_MH_, the authors obtained an effective mean thermal conductance *K*_Guard-MH_ between the MH and the guard heater system. *K*_Guard-MH_ was measured at 373 K < *T*_MH_ < 773 K and revealed a linear increase from 0.04 to 0.12 WK^−1^. The temperature distribution of the guard heater system and the MH was investigated by concurrent temperature measurements using 20 installed thermocouples. Inevitable temperature differences along the heat flow path from the MH to the sample, which occur even under nominally balanced temperature conditions of the MH and the guard heater system, amounted to maximum values between 1 and 18 K in dependence on *T*_MH_. Additional consideration of *K*_Guard-GHP_ allowed for quantification of the parasitic heat exchange within the GHP system and determination of the maximum heat flow uncertainty of the GHP apparatus of *u*(${\dot{Q}}_{\mathrm{GHP}}$) = 0.1–0.8% (*k* = 2) for optimal temperature settings of the guard heater system.

The given overview does not claim for completeness but indicates widespread incomplete awareness of uncertainties in TEM metrology. Often, unspecified uncertainty budgets reflect the current lack of reference and standardization, which is partly caused by the lack of appropriate TEM reference samples. The use of uncertified reference samples requires multiple experiments under repetitive conditions to determine measurement uncertainties according to official regulations given, for instance, by the GUM. Such time-consuming investigations are rarely performed, which leads to unnoticed or underestimated influencing parameters on TEM measurands. In particular, the statistic distribution and sensitivity coefficients of individual parameters are unknown or not well known for many employed TEM characterization devices. According to normative directives, such data build an indispensable basis to express individual uncertainty contributions and to quantify the overall combined uncertainty for any measurand. Compliance with approved guidelines for the expression of uncertainties gives access to traceable results for TEM properties with high confidence and enables stepwise improvements in metrological devices with respect to design, control functions, and evaluation procedures for better accuracy and reliability.

### 2.2. TEGMA—Thermoelectric Generator Measurement Apparatus

**Figure 2:**
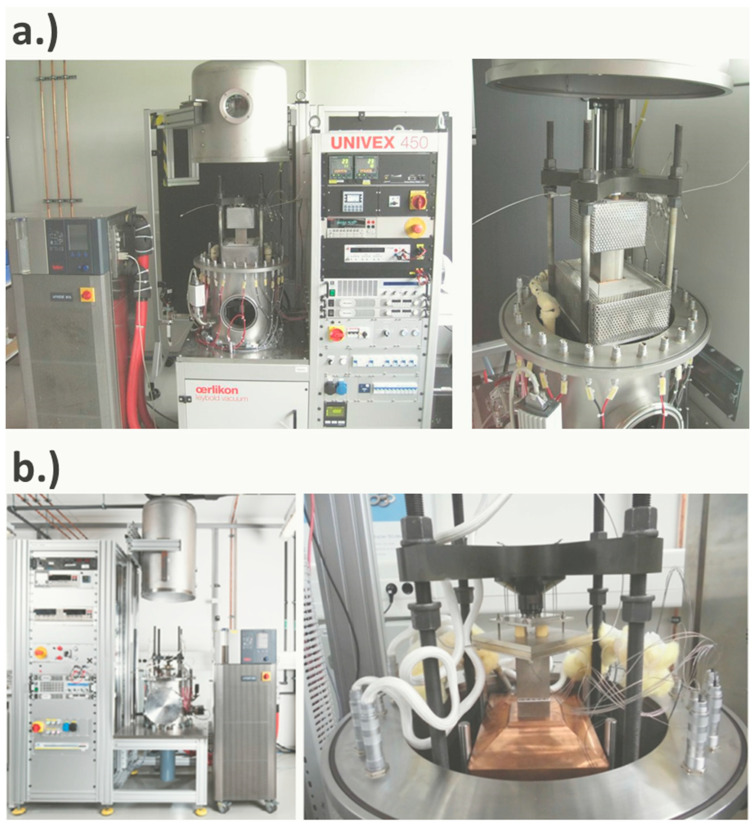
“A-TEGMA” (**a**) and “TEGMA” (**b**) devices for TEM characterization.

The A-TEGMA and TEGMA (Figure 2) are metrological devices for fully automated TEM characterization under variable boundary conditions (atmosphere, mechanical contact pressure, temperature, current flow). Although representing separate device setups, both facilities have been constructed similarly and comprise identical electronic instrumentation, with the only exception of components for heat flow determination. The reference principle is applied within the TEGMA, whereas the A-TEGMA offers additionally a GHP-based measurement. A comprehensive description of both facilities has been given in [51], which includes a detailed presentation of their instrumentation, measurement procedures, and analytic functions for the evaluation of TEM properties.

Both facilities comply with a uniform measurement protocol, which involves the setting of measurement boundary conditions at the beginning. First, the atmospheric (pressure of inert gas/vacuum) and the axial pressure, which is applied to the TEM sample, are set. Next, temperature conditions on both sides of the sample are adjusted by the setting of appropriate temperatures for the heater and the cooler. Measurements of TEM properties are conducted under thermal steady-state conditions. Thus, the temperatures of heaters, heat sinks, and exchangeable components within the measuring sections (HFM, heat exchangers, plates) are continuously recorded and analyzed during the transient phase of temperature adjustment. The required temperature stabilization time varies in dependence on the installed components and depends on their properties, such as geometry, thermal conductivity, and specific heat. For heat flow determination by means of the reference principle, the temperature stability is assessed by the remaining temperature drift at the hot and cold side of the TEM, which are typically limited to maximum values of 0.15 K min^−1^. This yields a usual total stabilization time in the order of 1 h. The GHP-based measuring system has to fulfil the same criterion but additionally with regard to the maximum temperature drift of the MH and all guard heaters, too. The intentionally weak thermal coupling between the MH and the guard heaters, which is caused by thermal insulation, and the thermal insulation between the guard heaters and their outer environment lead to much longer stabilization times of the GHP, reaching the order of 3 h. Independent of the method for heat flow determination, further temperature stabilization is needed after every variation of the electric current flow through the TEM due to an induced change in the heat balance of the measuring section, which is caused by the Peltier effect and the generated Joule heat inside the TEM. These current-related effects usually induce much smaller changes in heat flow and temperatures along the measuring section compared to the adjustment of new temperature set points for the heater or cooler, respectively. Consequently, compliance with maximum limits of the allowed temperature drift for thermal stability is usually reached after shorter stabilization times of approximately 10–20 min.

The functional characterization of TEMs follows a defined sequence after reaching thermal stability. This measurement procedure was discussed along with a description of applied characterization and evaluation methods for electric, thermal, and thermoelectric TEM properties in previous works [51,52]. It should be noted at this point that all relevant measurement signals are recorded after temperature stabilization for a period of typically 3–5 min. This elongated reading time is used to account for signal fluctuations, which can stem from noise, elusive offsets, or temperature fluctuations induced by the instrumentation for temperature control. The captured raw data for temperatures, voltages, and electric currents are forwarded to evaluation procedures for the calculation of fitting residuals from mathematical post-processing routines and distribution functions. These evaluation routines result in quantified standard deviations, sensitivity coefficients, and best estimates for particular measurands, building the basis for the expression of individual uncertainty contributions and combined uncertainties.

### 2.3. Round Robin Tests on TEM Metrology

#### 2.3.1. Sample Information

Commercial Bi_2_Te_3_-based TEMs from four manufacturers have been investigated in a previous study [52] concerning their functional stability, assessed by measurements of the internal electric resistance *R*_i_ during short- and long-term stability tests under temperature cycling. The most stable TEM type showed lowest changes over time Δ*R*_i_ = 0.25% (short-term test) and 2.43% (long-term test), respectively. Uncertainty budgets for the measurement of *R*_i_ have been evaluated and revealed a combined uncertainty of *u*(*R*_i_) = 2.97% (*k* = 2) for this module type. This confirmed the barely traceable degradation of module properties in the course of short- and long-term stability tests, since Δ*R*_i_ remained under the limit of *u*(*R*_i_). Additional tests revealed the lowest sensitivity of *R*_i_ against variations in the mechanical pressure for this module type (Δ*R*_i_p_ = −1.07% within a pressure interval of 1.5 MPa) and compared to other tested module types likewise a superior similarity (homogeneity) of *R*_i_ (1.21% < Δ*R*_i_h_ < 3.11% within a temperature interval of 100 K), which was investigated by comparative measurements on four TEMs. Table 1 summarizes additional information about this module type, which was selected for comparative samples of the RR test. Three new modules of this type were forwarded to the RR after the abovementioned test series.

**Figure 3:**
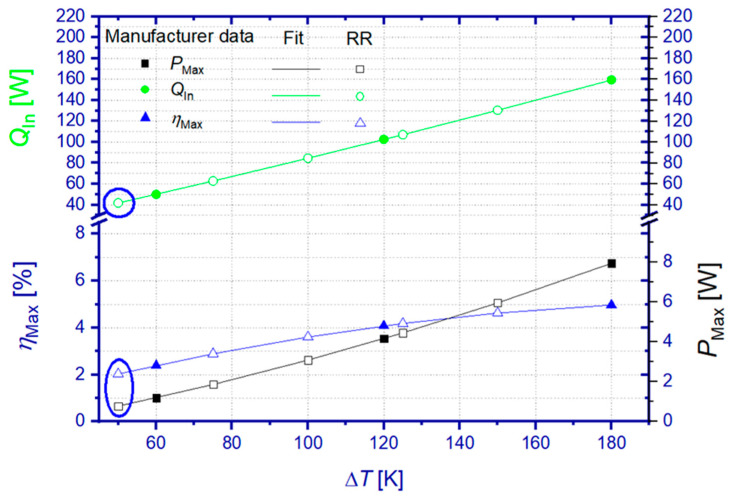
Manufacturer specifications (full symbols) on maximum power output, incident heat flow at maximum efficiency conditions, and maximum efficiency in dependence on the applied temperature difference across the TEM. The manufacturer specifications refer to a cold side temperature of *T*_C_ = 50 °C. Manufacturer’s reference data (blank symbols) for comparison of RR results were derived from polynomial approximations (lines) of given module specifications. Extrapolated reference values obtained from approximation functions of manufacturer data are limited to the lowest Δ*T =* 50 K and indicated by blue circles.

Results of the RR have been compared to TEM specifications from the supplier datasheet. The comparative values were calculated from polynomial approximation of the manufacturer references, since the original data referred to other hot side temperatures than the ones during the RR. Except for the lowest temperature difference during the RR test (Δ*T* = 50 K), all manufacturer’s comparative data have been obtained from interpolated values of respective approximation functions. Figure 3 visualizes the TEM specifications of the manufacturer, approximation functions, and derived reference values representative for temperature boundary conditions of the RR.

#### 2.3.2. Participants, Organization, and Test Program of the Round Robin Test

An international RR campaign was organized among twelve laboratories from seven countries in Asia, North America, and Europe in order to review the quality of TEM measurements and to derive a representative estimate on the comparability of employed measurement techniques. The RR was conceived as a blind study. An exception is made for DLR results, which are disclosed as measurements by Lab3a/3b (A-TEGMA) and Lab3c (TEGMA), since specified uncertainty budgets are needed to assess deviations among RR measurements. Measurements conducted at DLR contain heat flow results obtained by the GHP method (Lab3a–A-TEGMA) and the reference principle (Lab3b–A-TEGMA/Lab3c–TEGMA).

All participants confirmed the operation of custom-built devices for TEM characterization. Commercial measurement devices, although available in the market, did not participate in the RR. Detailed information on construction, instrumentation, and underlying measurement and evaluation protocols was not available from the participants to an extent, which could allow for a thorough case analysis of observed deviations of measurement results.

Due to the limited execution time of the RR, the participants were split into three groups. Each of the three selected TEM samples was assigned to one group, as shown in Table 2. Each TEM shipment was additionally equipped with fresh graphite foils (Dr. Fritsch Gerätebau GmbH, 200 µm thickness), which were taken from one and the same batch in order to ensure the most similar thermal coupling conditions at the hot and cold sides of the TEMs. Modules were resent after each measurement to the managing laboratory of the RR at DLR. This allowed for repeated visual inspection of the module integrity and short room temperature tests of *R*_i_ in order to exclude intermediate module damage and to ensure the best possible comparability for following measurements.

Participating laboratories were requested to conduct measurements under vacuum conditions. Each laboratory was asked to conduct one temperature cycle (up and down) at the hot side of the TEM, covering five stabilized temperature points (*T*_H_ = 100/125/150/175/200 °C) while keeping the cold side temperature constant (*T*_C_ = 50 °C). From Laboratory 8, no results on the heat flow and efficiency were available but only for the maximum power output during the heating sequence of the temperature cycle. Moreover, Laboratory 12 delivered data only from the heating sequence. With the exception of the supplied graphite foils, every laboratory was requested to conduct measurements similarly to routine tests with standard components, usually used instrumentation and evaluation protocols, and with typically applied settings for stabilization times and the density of setpoints for the variation of the electric current flow. In order to involve as many participants as possible, an axial pressure of 1 MPa was specified for the RR tests, since higher values could not be applied by some of the participating custom-built characterization facilities.

The maximum power output, the incident heat flow at the hot side of the TEM (at maximum efficiency operation conditions), and the maximum efficiency had to be determined at predefined boundary conditions. In the case of heat flow measurements at the cold side of a TEM, the electric power output was added to the measured heat flow to conclude on the incident heat flow. Although participants were asked to provide heat flow data for maximum efficiency conditions, some laboratories (Lab 4, 6, 10) sent only those results for maximum power output conditions.

Generally, the provided data by all laboratories showed slight differences for the effective hot and cold side temperatures in the order of a few K due to varying approaches for temperature stabilization and individual contributions of thermal transfer resistances from components along the heat transmission paths of the measuring sections. In order to eliminate the effect of differing temperature conditions on the comparison of RR data, every temperature-dependent data set was fitted individually for the heating and cooling sequence by a fourth-order polynomial function. Based on these approximation functions, interpolated values have been calculated for heating and cooling curves of each measurand at uniform temperature intervals. Mean values have been determined from interpolated data points of the approximated heating and cooling curves and finally entered the comparison of RR results. Generally, the standard deviation of the resulting mean values can be interpreted either as an indication of TEM instability (divergence of results from heating and cooling sequence), minor reproducibility of measurement conditions during a temperature cycle (variation of temperatures or contact pressure), or persisting measurement uncertainties. Since no significant TEM degradation could be observed before, during, and after successive RR tests, data differences between heating and cooling sequences are linked most likely to uncertainties and/or changing measurement conditions in the course of the applied temperature cycle. A summary on the standard deviation of interpolated mean values from heating and cooling curves can be found for each tested TEM in the Supplementary Information S1 within Figures S1–S3.

## 3. Results and Discussion

### 3.1. Uncertainty of TEGMA Power Measurement

The power output *P* of a TEM is determined according to Equation (3) and based on measurements of the current flow *I* and the terminal voltage *V* of a TEM. The combined uncertainty *u*(*P*) considers individual uncertainty contributions of both input variables, which are combined in a geometric sum. Every contribution equals the product of a standard measurement uncertainty *u* and the sensitivity coefficient *c* of the respective input variable.

(9) $$u\left(P\right)=\sqrt{c_{I}^{2}u{\left(I\right)}^{2}+c_{V}^{2}u{\left(V\right)}^{2}}$$

(10) $$c_{I}=\frac{dP}{dI}=V$$

(11) $$c_{V}=\frac{dP}{dV}=I$$

Here, *I* and *V* denote best estimates of both measured values. Best estimates equal expectation or mean values from the repetitive measurements at given and nominally constant TEM boundary conditions. The standard uncertainty of a voltage measurement *u*(*V*) relates either to the distribution width of captured data or to the measurement uncertainty of the measurement hardware (e.g., a digital multimeter—DMM), whichever is greater. Typically, result distributions exceed the expectable uncertainty range of a DMM due to the presence of signal noise, offsets, or temperature fluctuations. Assuming a Gaussian data distribution, the full width at half maximum value (FWHM) enables the determination of the standard measurement uncertainty by means of Equation (12) [102].

(12) $$u=\frac{FWHM}{2\;\sqrt{2\;\ln2}}$$

Alternatively, the empiric standard deviation s is taken for specification of the uncertainty in case of random data distributions [50,103].

(13) $$s=\sqrt{\frac{1}{N-1}\overset{N}{\underset{i=1}{\sum }}{\left(V_{j}-\bar{V}\right)}^{2}}$$

with N being the number of data points within a set (in this study, N = 10), $V_{j}$ as the *j*th reading, and $\bar{V}$ as the mean value of the result distribution. The resulting standard measurement uncertainty *u* is calculated from s according to Equation (14) [50,103].

(14) $$u=\frac{s}{\sqrt{N}}$$

The current *I* is deduced from the voltage drop $V_{\mathrm{Ref}}$ over a high-precision shunt resistor $R_{\mathrm{Ref}}$ = 0.1 Ω (PBV0.1, Isabellenhütte), which has a tolerance of ±0.5% [104].

(15) $$I=\frac{V_{\mathrm{Ref}}}{R_{\mathrm{Ref}}}$$

The combined uncertainty *u*(*I*) is calculated by a sub-model, which is based on Equation (15). Consequently, *u*(*I*) accounts for uncertainty contributions from the voltage measurement and the tolerance *u*($R_{\mathrm{Ref}}$) of the shunt resistor $R_{\mathrm{Ref}}$.

(16) $$u\left(I\right)=\sqrt{c_{VRef}^{2}u{\left(V_{\mathrm{Ref}}\right)}^{2}+c_{R}^{2}u{\left(R_{\mathrm{Ref}}\right)}^{2}}$$

(17) $$c_{VRef}=\frac{dI}{dV_{\mathrm{Ref}}}=\frac{1}{R_{\mathrm{Ref}}}$$

(18) $$c_{R}=\frac{dI}{dR_{\mathrm{Ref}}}=-\frac{V_{\mathrm{Ref}}}{{\left(R_{\mathrm{Ref}}\right)}^{2}}$$

Again, partial derivatives of the underlying measurement instruction (Equation (15)) are taken for the calculation of individual sensitivity coefficients. $V_{\mathrm{Ref}}$ is the mean value of all voltage readings and the standard uncertainty *u*($V_{\mathrm{Ref}}$), which is determined by the above-specified procedure, enabling the calculation of the uncertainty contribution of the voltage measurement. The specified value of the shunt resistor $R_{\mathrm{Ref}}$ = 0.1 Ω, while its tolerance equals its corresponding standard uncertainty *u*($R_{\mathrm{Ref}}$) = 0.5 mΩ.

The power output is usually measured at discrete current setpoints, which do not necessarily match with the optimum operation points of the TEM for maximum efficiency or maximum power output, respectively. In order to obtain the maximum power output *P*_max_, the power output *P* is initially approximated by a parabolic function of *I*.

(19) $$P=a\cdot I^{2}+b\cdot I+c$$

Herein, *a*, *b*, and *c* denote the coefficients of the approximated power parabola function. The optimum current flow $I_{\mathrm{opt},P}$ for maximum power output is derived by setting the partial derivative function to zero.

(20) $$\frac{dP}{dI}=2a\cdot I_{\mathrm{opt},P}+b=0$$

(21) $$I_{\mathrm{opt},P}=\frac{-b}{2a}$$

The maximum power output *P*_max_ corresponds to *P*($I_{\mathrm{opt},P}$).

(22) $$P_{\max}=P\left(I_{\mathrm{opt},P}\right)=c-\frac{b^{2}}{4a}$$

As stated above, every measurement of *P* is subjected to signal fluctuations. The resulting uncertainty of *P*_max_ can be determined from the standard uncertainties of the coefficients of the parabolic approximation *u*(*a*), *u*(*b*), and *u*(*c*) and corresponding sensitivity coefficients.

(23) $$u\left(P_{\max}\right)=\sqrt{c_{a}^{2}u{\left(a\right)}^{2}+c_{b}^{2}u{\left(b\right)}^{2}+c_{c}^{2}u{\left(c\right)}^{2}}$$

(24) $$c_{a}=\frac{dP_{\max}}{da}=\frac{b^{2}}{4a^{2}}$$

(25) $$c_{b}=\frac{dP_{\max}}{db}=\frac{-b}{2a}$$

(26) $$c_{c}=\frac{dP_{\max}}{dc}=1$$

Based on the exemplary characterization results of DLR (Lab 3a/3b) obtained during the RR on sample TEM 3, the following discussion shall demonstrate the quantitative evaluation of *u*(*P*) and *u*(*P*_max_) for a single temperature point. Additional information on underlying data for best estimates, sensitivity coefficients, and individual uncertainty contributions is given for every input variable and every tested boundary condition in the Supplementary Information S2 within Table S1.

**Figure 4:**
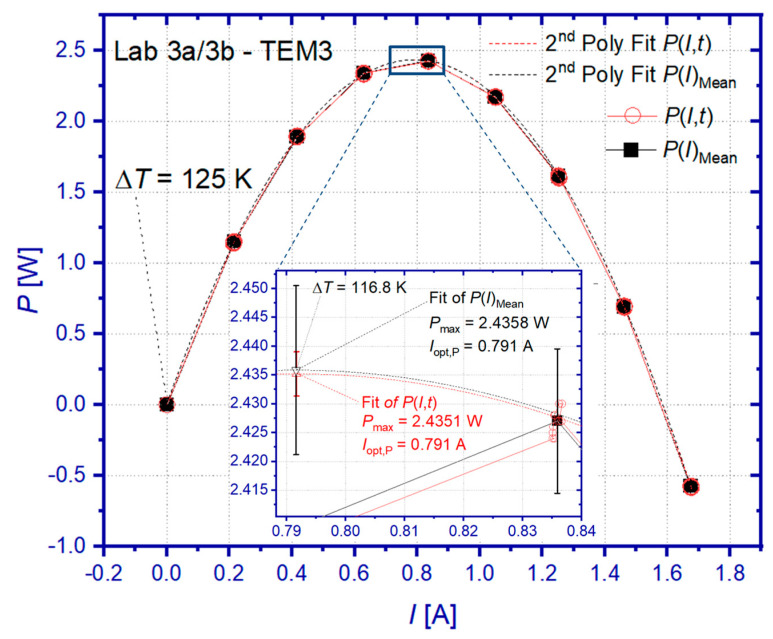
Power output *P* of TEM3, measured at DLR (Lab 3a/3b) in dependence on current flow *I* for Δ*T* = 125 K at open-loop conditions. *P*(*I*,*t*) represents raw data for nominally stable boundary conditions. *P*(*I*)_Mean_ is calculated as an average of *P*(*I*,*t*) for each current value. The shown error bar of *P*(*I*)_Mean_ corresponds to the uncertainty *u*(*P*(*I*,*t*)) (*k* = 1) according to Equation (9). Both *P*(*I*,*t*) and *P*(*I*)_Mean_ are input to parabolic approximations (dashed lines). The inset indicates the maximum power points determined from approximations of *P*(*I*,*t*) (open red triangle) and *P*(*I*)_Mean_ (open black triangle). Error bars of both maximum power points correspond to uncertainties *u*(*P*_max_) (*k* = 1) according to Equation (23).

Figure 4 shows the measured dependence *P*(*I*) of the sample TEM3 for Δ*T* = 125 K at open-loop condition (*I* = 0). Generally, Δ*T* is initially stabilized at the TEM for *I* = 0. Both the heater and cooler temperature setpoints are not changed during the following eight current steps, which yields a successive decrease in Δ*T* with increasing current flow due to the Peltier effect. For the case considered, Δ*T* decreased from 125 K at open-loop condition to 116.8 K at optimum current for maximum power output (Figure 4 inset). After every current change, the system is allowed to stabilize with respect to temperatures at the TEM. The electric current and the terminal voltage are measured after stabilization during an observation time of at least 3 min, which provides the shown data on *P*(*I*,*t*). As can be seen from the inset in Figure 4, *P*(*I*,*t*) scatters due to fluctuations in recorded current and voltage values. The mean value *P*(*I*)_Mean_ is calculated from all values of *P*(*I*,*t*) at a given current setpoint, along with standard uncertainties and sensitivity coefficients for the quantification of *u*(*P*) according to Equation (9). The resulting *u*(*P*) is indicated within the inset of Figure 4 by an error bar for *P*(*I*)_Mean_ at a current of approximately 0.835 A. More details on the current dependence of *u*(*P*) can be found in Figure S4 within the Supplementary Information S2. However, the average absolute uncertainty *u*(*P*)_avg_ (*k* = 2), which is calculated as the mean value of all *u*(*P*) within a tested current interval, increases with increasing temperature difference from 5.27 up to 24.26 mW, whereas corresponding relative values decrease from 2.33% for the lowest Δ*T* to 1.25% at the highest Δ*T* (Table 3).

The uncertainty contribution of the current measurement $c_{I}$⋅*u*(*I*) dominates *u*(*P*) in the low and medium current range, while the uncertainty contribution of the voltage measurement $c_{V}$⋅*u*(*V*) becomes more significant at higher current flow (Figure S5). The uncertainty *u*(*I*) is mainly determined by the uncertainty contribution due to the tolerance of the shunt resistor $c_{R}$⋅*u*($R_{\mathrm{Ref}}$), which exceeds the contribution of the voltage measurement at the shunt resistor $c_{VRef}$⋅*u*($V_{\mathrm{Ref}}$) significantly (Figure S6) and almost over the entire current range except at lowest current. Additional information on the calculation of *u*(*P*) can be found in Table S1, which summarizes underlying data for every input variable in dependence on Δ*T*. It should be mentioned at this point that the temperature-averaged relative uncertainty *u*(*P*)_avg_ = 0.86% (*k* = 1) determined by this study is in good agreement with 0.85% of power deviation, which was reported in a previous work about an inter-laboratory test on a Ni-based prototype TEM for prospective use as a high-temperature metrological reference sample [49].

Data of *P*(*I*,*t*) and *P*(*I*)_Mean_ are used for parabolic approximations, which each provide coefficients *a*, *b*, and *c* according to Equation (19), allowing for the determination of the optimum current flow $I_{\mathrm{opt},P}$ according to Equation (21) and the maximum power output *P*_max_ according to Equation (22). The inset of Figure 4 reveals only marginal differences in *P*_max_ and $I_{\mathrm{opt},P}$ obtained from both approximations but confirms the lower uncertainty *u*(*P*_max_) for the approximation of *P*(*I*,*t*) compared to *P*(*I*)_Mean_. A detailed comparison of results, standard uncertainties, and sensitivity coefficients can be found for both input data sets in dependence on Δ*T* within Table S2.

**Figure 5:**
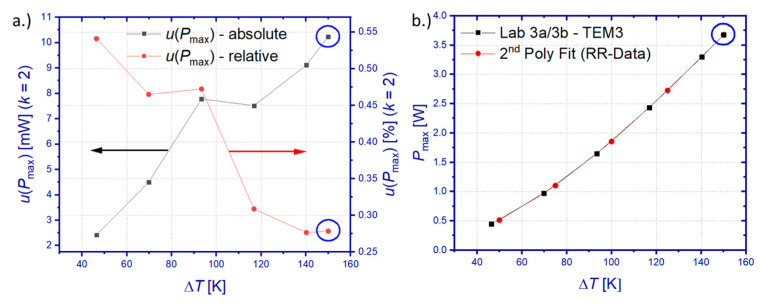
The absolute and relative uncertainty *u*(*P*_max_) (*k* = 2) (**a**) and *P*_max_ (**b**) are shown in dependence on Δ*T*. *P*_max_ has been determined from a parabolic approximation of *P*(*I*,*t*) according to Equation (19), while *u*(*P*_max_) was determined from Equation (23). In order to achieve comparability of RR results, DLR data were interpolated for Δ*T* = 50, 75, 100, and 125 K and extrapolated for Δ*T* = 150 K. A linear fit was used for *u*(*P*_max_), whereas *P*_max_ has been approximated by a parabolic fit. Extrapolated values are indicated by blue circles.

Figure 5a shows the combined uncertainty for the determination of *P*_max_. Similarly as for *u*(*P*)_avg_, likewise *u*(*P*_max_) increases absolutely but shows a decreasing trend of relative values with increasing temperature difference over the TEM. The relative *u*(*P*_max_) lies within 0.27% < *u*(*P*_max_) < 0.54% (*k* = 2) for the tested temperature range. Both the absolute and relative *u*(*P*_max_) are displayed in Figure 5a for a confidence interval of 95%. In order to achieve the highest possible comparability of RR results, DLR data for *u*(*P*_max_) and *P*_max_ were interpolated for Δ*T* = 50, 75, 100, and 125 K, whereas extrapolated for Δ*T* = 150 K. Here, a piecewise-linear interpolation has been used for *u*(*P*_max_), whereas a parabolic approximation was used for *P*_max_.

### 3.2. Uncertainty of TEGMA Heat Flow Measurement

**Figure 6:**
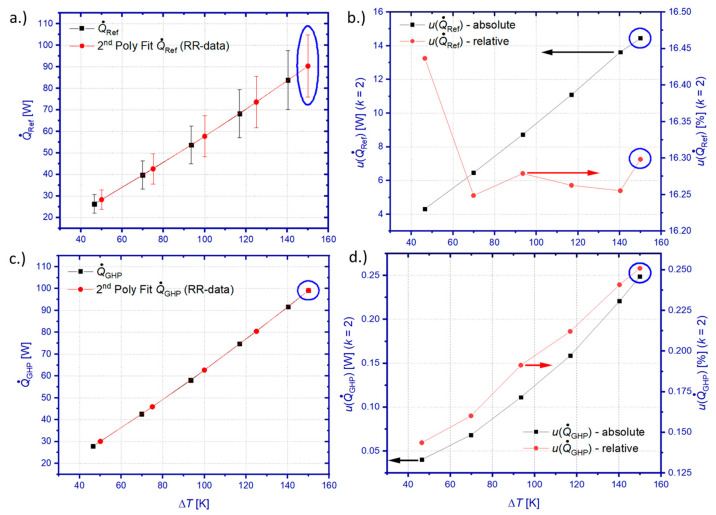
Heat flow measurement results (best estimates) of the reference principle (**a**) and the guarded hot plate (**c**) in dependence on Δ*T*. Combined uncertainties of heat flow determination (*k* = 2) are displayed as absolute and relative values for the reference principle (**b**) and the guarded hot plate (**d**). For details on the determination of uncertainties refer to Supplementary Information S3. In order to achieve comparability of RR heat flow results, DLR data were interpolated for Δ*T* = 50, 75, 100, and 125 K and extrapolated for Δ*T* = 150 K. Parabolic fits have been used for the approximation of uncertainties *u*(${\dot{Q}}_{\mathrm{Ref}}$) and *u*(${\dot{Q}}_{\mathrm{GHP}}$) and heat flow results ${\dot{Q}}_{\mathrm{Ref}}$ and ${\dot{Q}}_{\mathrm{GHP}}$. Extrapolated values of these fits are indicated by blue circles.

Equations (7) and (8) are measurement functions for heat flow determination by means of the reference principle and the GHP method, respectively. The methodology, analytic description, and discussion of individual uncertainty contributions for the expression of combined uncertainties *u*(${\dot{Q}}_{\mathrm{Ref}}$) and *u*(${\dot{Q}}_{\mathrm{GHP}}$) have been reported in a previous study, which was conducted on a comparative sample made from a thermal reference material [51]. The underlying approach is recapitulated for both characterization methods within Supporting Information S3 of this article, together with current data on sensitivity coefficients and uncertainty contributions obtained by this study on a TEM sample. Figure 6 summarizes best estimates (${\dot{Q}}_{\mathrm{Ref}}$/${\dot{Q}}_{\mathrm{GHP}}$) and results for *u*(${\dot{Q}}_{\mathrm{Ref}}$) and *u*(${\dot{Q}}_{\mathrm{GHP}}$) in dependence on the temperature boundary conditions.

Both methods reveal a monotonous increase in absolute values of heat flow uncertainties with increasing Δ*T*. The uncertainty of the reference principle *u*(${\dot{Q}}_{\mathrm{Ref}}$) lies between 4.32 and 14.45 W (*k* = 2), which corresponds to an almost constant relative uncertainty slightly higher than 16% for the entire temperature interval. The main contributor to *u*(${\dot{Q}}_{\mathrm{Ref}}$) is the uncertainty of the thermal conductivity of the used HFM, which exceeds other contributions such as the temperature gradient measurement (Figure S7) and the uncertainty of the cross-section of the HFM over the entire temperature range (Figure S8, Table S3). The main source of uncertainty of the GHP method is given by the thermal crosstalk between the MH and its guard heater system [81,82,83]. The heat flow, which is generated by the MH, causes an inevitable temperature difference Δ*T*_MH_ = *T*_MH_ − *T*_HC_ along the heat flow path inside the GHP from the MH to the hot coupling surface of the GHP, which has the temperature *T*_HC_. This temperature spread prevents the adjustment of isothermal conditions between the MH and the guard heater, which is used to surround this heat flow path and to shield it thermally. The respective heat exchange can be minimized if this guard heater is set to a temperature between the maximum (*T*_MH_) and minimum (*T*_HC_) temperature inside the GHP system. However, in a worst-case consideration, one has to assume Δ*T*_MH_ as the maximum temperature deviation between the MH and this guard heater. This maximum temperature deviation and the effective thermal conductance between the guard heater system and the MH, which was determined experimentally (Figure S9), build the basis for the expression of the uncertainty of the GHP method *u*(${\dot{Q}}_{\mathrm{GHP}}$). The uncertainty *u*(${\dot{Q}}_{\mathrm{GHP}}$) is considerably lower than *u*(${\dot{Q}}_{\mathrm{Ref}}$) and lies in this study between 0.04 and 0.25 W, which corresponds to a relative uncertainty between 0.14% and 0.25%. A previous study on a thermal reference material [51] revealed slightly higher values of 0.2% < *u*(${\dot{Q}}_{\mathrm{GHP}}$) < 0.75% than this study. The reduced uncertainty of this study can be explained by the lowered heat flow through the tested TEM and therefore lowered Δ*T*_MH_ compared to the previously tested reference material.

Best estimates of heat flow data of the GHP method show systematically higher values over the entire temperature range compared to the outcome of the reference principle. The deviation between ${\dot{Q}}_{\mathrm{GHP}}$ and ${\dot{Q}}_{\mathrm{Ref}}$ scales almost linearly with Δ*T* and lies between 1.61 and 7.93 W, which corresponds to 5.7% and 8.7% of ${\dot{Q}}_{\mathrm{GHP}}$, respectively. It should be noted that this deviation is still within the uncertainty budget of the reference principle, similarly as was observed by other works [51,99]. The deviation of both methods is caused in this study by the configuration of the measuring section, which made use of an HFM between the GHP and the hot side of the tested TEM. This opens the possibility of parasitic heat losses along the HFM, which yields a systematically higher heat flow by the GHP method compared to the reference principle. Better accordance of both measurement methods was achieved for a cold side heat flow measurement according to the reference principle, effectively yielding configurations with direct coupling between the GHP and the sample. Tests of such configurations have been described by previously conducted studies [49] and [51], which yielded deviations between ${\dot{Q}}_{\mathrm{GHP}}$ and ${\dot{Q}}_{\mathrm{Ref}}$ lower than 1.5% and 1.14%, respectively.

### 3.3. Uncertainty of TEGMA Efficiency Measurement

Equation (5) describes the TEM efficiency as a function of power output *P* and incident heat flow ${\dot{Q}}_{\mathrm{In}}$ at the hot side of the TEM. The combined measurement uncertainty for the TEM efficiency *u*(*η*), which depends on sensitivity coefficients and standard measurement uncertainties (*k* = 1) for both input variables, can be expressed by the following equation.

(27) $$u\left(\eta \right)=\sqrt{c_{P}^{2}u{\left(P\right)}^{2}+c_{QIn}^{2}u{\left({\dot{Q}}_{\mathrm{In}}\right)}^{2}}$$

(28) $$c_{P}=\frac{d\eta }{dP}=\frac{1}{{\dot{Q}}_{\mathrm{In}}}$$

(29) $$c_{QIn}=\frac{d\eta }{dQ_{\mathrm{In}}}=\frac{-P}{{\left({\dot{Q}}_{\mathrm{In}}\right)}^{2}}$$

**Figure 7:**
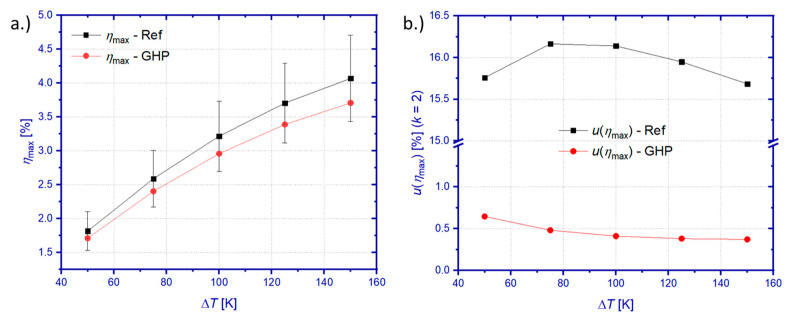
Best estimates for the maximum efficiency *η*_max_ obtained from Equation (5) with data on power output and heat flow from the reference principle and the GHP method, respectively (**a**). The error bars, which are actually only visible for the reference principle, give combined uncertainties of the maximum efficiency *u*(*η*_max_) (*k* = 2). These uncertainties have been determined for both heat flow measurement methods using Equation (27) (**b**).

Sensitivity coefficients $c_{P}$ and $c_{QIn}$, best estimates *P* and ${\dot{Q}}_{\mathrm{GHP}}$/${\dot{Q}}_{\mathrm{Ref}}$ (as ${\dot{Q}}_{\mathrm{In}}$), and standard measurement uncertainties *u*(*P*) and *u*(${\dot{Q}}_{\mathrm{GHP}}$)/*u*(${\dot{Q}}_{\mathrm{Ref}}$) have been calculated for conditions of maximum efficiency operation and used for the determination of *η*_Max_ and *u*(*η*_Max_). Figure 7 summarizes these results.

The higher heat flow measured by the GHP method yields lower maximum efficiency compared to the reference principle. However, similarly as for the heat flow results, the best estimate *η*_max_ of the GHP method is still within the uncertainty limit of results from the reference principle (Figure 7a). Considerable differences in heat flow measurement uncertainties are passed on to the uncertainty of the maximum efficiency (Figure 7b). The uncertainty contribution of the heat flow measurement exceeds the contribution of the power measurement by orders of magnitude (Figure S10), which yields a combined uncertainty of 0.37% < *u*(*η*_max_) < 0.64% for the GHP method and 15.68% < *u*(*η*_max_) < 16.12% for the reference principle. The uncertainty *u*(*η*_max_) of the GHP method is strictly only valid for direct coupling between the GHP and the TEM, since parasitic heat losses of a hot side HFM have not been taken into account.

### 3.4. Results of the Round Robin

#### 3.4.1. Maximum Power Output

**Figure 8:**
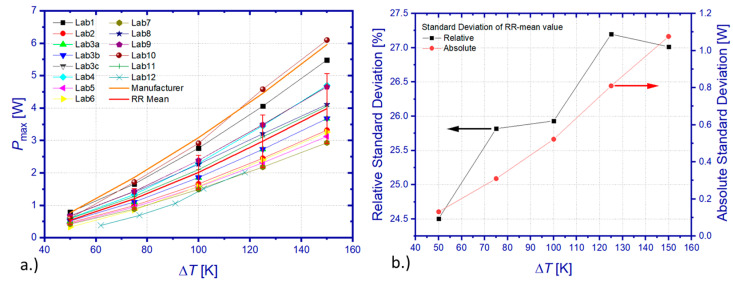
RR results for maximum power output *P*_max_ in dependence on the temperature difference Δ*T* in comparison to manufacturer specifications (**a**). The temperature-dependent mean value (RR mean) and its corresponding standard deviation have been calculated from all data sets. The absolute standard deviation is indicated as error bars (**a**) and shown separately together with the relative standard deviation (**b**).

The participants were requested to derive the maximum power output *P*_max_ from their usually employed procedures by means of approximations of captured power parabola curves *P*(*I*) or evaluation of *I*/*V* characteristics. Figure 8a summarizes all results for the maximum power output *P*_Max_, which have been obtained on all tested TEMs. Generally, results show similar temperature characteristics of *P*_Max_ but are subjected to significant deviations from each other and, in most cases, to manufacturer specifications, too. Calculation of the mean value of *P*_Max_ from all data sets (RR mean) revealed a standard deviation, which increases monotonously from 0.131 to 1.07 W within the tested temperature interval (Figure 8b). This corresponds to a relative standard deviation between 24.5% and 27.2% of the respective RR mean values of *P*_Max_. Comparison between the RR mean and the manufacturer’s reference data reveals a deviation between 28% and 35%. The deviation between the RR mean and manufacturer’s reference data is smallest for the lowest Δ*T* and increases with a rising temperature difference.

Deviation of power measurements can be principally caused by varying or uncertain boundary conditions and/or lacking accuracy of applied fitting routines during post-processing of the *I*/*V* characteristic or power curves. Apparently, the deviation among RR data for *P*_max_ exceeds a level that could be expected from previous studies on the power sensitivity of this module type against changes in the mechanical pressure (Δ*R*_i_ = 0.71%/MPa). The deviation of *P*_max_ due to uncertainties of set temperature conditions could potentially explain the power differences at lower Δ*T* but can hardly cause the observed level of deviation at higher values of Δ*T*. For at least three laboratories (Lab 2, 4, 8), the evaluation of submitted measurement data gave evidence of a too low number or unsuitable setting of electrical current values, which either ranged within an interval close to open-loop or short-circuit conditions only, while omitting measurements near the optimum operation conditions close to half of the short-circuit current. Consequently, extrapolation of power output data to the optimum current for *P*_max_ might be overlaid then by considerable uncertainties. Other laboratories have either used more than 20 test points of the electric current (Lab 2) or applied a continuous current sweep (Lab 10). Since no further information was given on employed stabilization times during changes of the electric current flow, both laboratories possibly provided measurement data for the power output from transient temperature conditions.

**Figure 9:**
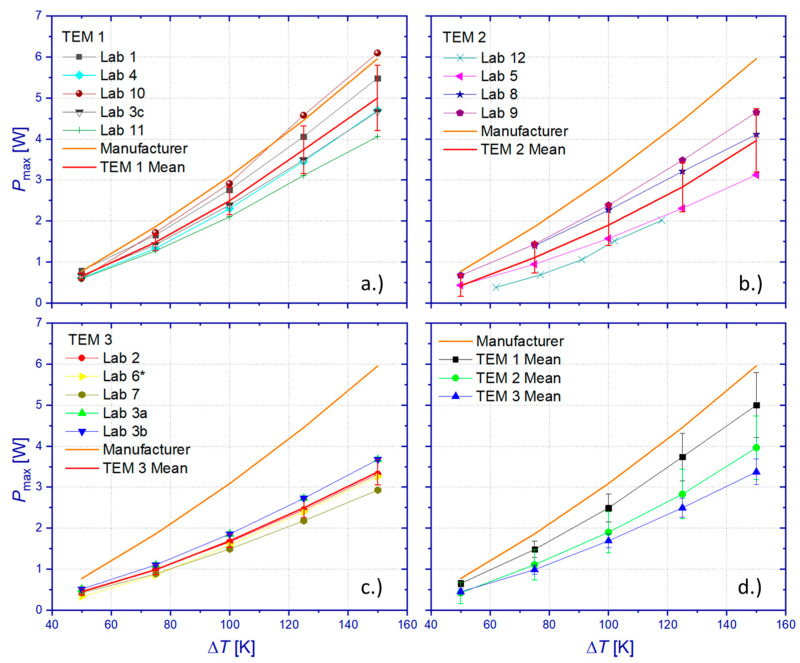
RR results for maximum power output *P*_max_ in dependence on the temperature difference Δ*T*. The results are shown separately for measurements on TEM1 (**a**), TEM2 (**b**), and TEM3 (**c**). Mean values and their corresponding standard deviation, which is displayed as error bars, have been calculated from all data sets of each TEM. Manufacturer specifications are shown for comparison with the mean values of the tested TEMs (**d**).

Another cause for the deviation of measured output power is given by different module properties due to manufacturing tolerance or due to intermediate module degradation. Figure 9a–c show TEM-specific results of *P*_max_. This comparison indicates a higher scatter of module properties compared to previous studies on the variation of the internal electric resistance *R*_i_, which was assessed on four TEMs of the same type and which was in the range of 1.21% < Δ*R*_i_ < 3.11%. The comparison of mean values among the TEM points to systematic differences in *P*_max_, which is highest for TEM 1 and lowest for TEM 3 (Figure 9d). This observation makes it difficult to exclude manufacturing tolerance as contributing to the large scatter of the RR test. However, TEM-specific relative standard deviations give likewise elevated values (TEM 1: 12.5–15.9%, TEM 2: 19.5–62.4%, TEM 3: 9.3–17.3%). A comparison between TEM-specific mean RR data for *P*_max_ and manufacturer specifications reveals maximum relative deviations of 19.2% (TEM 1), 46.6% (TEM 2), and 46.9% (TEM 3). Data comparison between participants of the RR and DLR results shows that only a single laboratory (TEM 1: Lab 4) obtained results for *P*_max_ within the uncertainty limit of measurements by DLR (Lab 3c). Results from Lab 10 showed good accordance with manufacturer specifications but ranged beyond the uncertainty limit of DLR measurements. It is worth noting that the manufacturer specifications for *P*_max_ could not be confirmed by DLR measurements on TEM 1 and TEM 3.

#### 3.4.2. Heat Flow at Maximum Efficiency Operation

Various methods have been used by RR participants for heat flow determination. Most laboratories applied the reference method using an HFM either on the hot or cold side of the TEM. Except for Lab 2, which has manufactured specific new heat exchanger parts with an adapted cross-section of 40 × 40 mm^2^, no further details have been revealed by other laboratories about the material choice, the geometry, or the sensor instrumentation of the employed HFMs. Two laboratories (Lab 1 and 3a) applied an absolute heat flow measurement by active GHP. Lab 10 applied an absolute heat flow determination using passive thermal insulation only. In order to consider occurring heat losses, temperature-dependent correction factors have been applied by Lab 10, which were previously determined from FEM simulations and analytic calculations for generic cases reflecting exemplary measurement conditions for TEM but not specifically those applied for measurements of the samples of the RR.

**Figure 10:**
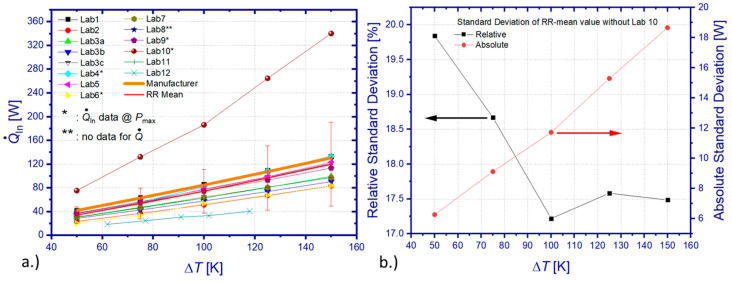
RR results for incident heat flow ${\dot{Q}}_{\mathrm{In}}$ at conditions of maximum efficiency operation in dependence on the temperature difference Δ*T* (**a**). The temperature-dependent mean value (RR mean) and the standard deviation have been calculated from all data sets for the left diagram. The standard deviation is indicated here by error bars for the RR mean. This diagram contains additionally a comparison to manufacturer specifications. The absolute and relative standard deviations have been calculated, excluding data from Lab 10 (**b**).

Figure 10a shows the comparison between all heat flow measurement results of the RR. The overall standard deviation of all data sets from the mean value scales linearly with Δ*T* from 14.1 to 70.7 W, which equals a relative deviation between 41.6% and 59.2%. In view of the significant deviation of results from Lab 10, heat losses of the applied absolute method have been apparently underestimated, yielding an insufficient correction of measurement data. However, even omitting results from this particular laboratory, the resulting standard deviation of the heat flow mean value (RR mean) still ranges from 6.2 to 18.6 W, corresponding to 19.8% and 17.48% of the mean value (Figure 10b) within the tested temperature interval. Differences among RR results related to heat flow determination for operation conditions of maximum power output or maximum efficiency cannot contribute significantly to these deviations since TEM properties barely vary by more than 1% between these operation points. Furthermore, a comparable level of deviation could be observed for *P*_max_, which was determined by all participants according to their usually applied procedures and without doubt concerning the chosen operation point. Comparing the RR mean (excluding Lab 10) with manufacturer reference data reveals a deviation between 24.1% and 18.1%. A surprising finding is that, contrary to the maximum power output, the relative heat flow deviation between the RR mean (from all data sets) and the manufacturer’s specification is highest for a small Δ*T*.

**Figure 11:**
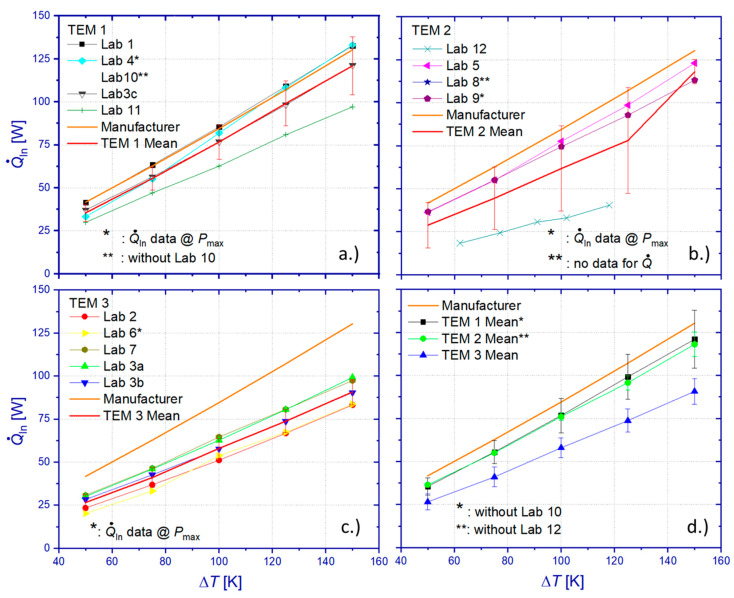
RR results for incident heat flow ${\dot{Q}}_{\mathrm{In}}$ at maximum efficiency in dependence on the temperature difference Δ*T*, shown separately for TEM1 ((**a**) without Lab 10), TEM2 (**b**), and TEM3 (**c**). Mean values and standard deviation displayed by error bars have been calculated excluding certain data sets as indicated and compared to manufacturer specifications. Comparison among the mean values for the TEM is shown in (**d**).

A TEM-specific evaluation of heat flow data (Figure 11a–c) yields a relative standard deviation of individual mean values of 13.8–14% (TEM 1), 1–5.9% (TEM 2 without Lab 12), and 17–8.2% (TEM 3), respectively. The relative standard deviation is increasing with Δ*T* for all tested TEM, probably mainly due to an increase in unnoticed heat losses at elevated temperatures by radiative heat exchange.

Contrarily to the observed scatter of *P*_max_, which indicated a dissimilarity of the tested modules (Figure 9d), the TEM-specific comparison of resulting mean values for the heat flow ${\dot{Q}}_{\mathrm{in}}$ (Figure 11d) revealed similar mean values within the limits of the standard deviations for TEM 1 and TEM 2. A comparison to manufacturer specifications showed maximum relative deviations of 14.8% (TEM 1), 12.6% (TEM 2), and 36.3% (TEM 3). Results of Lab 1 and Lab 4 show very good accordance with manufacturer specifications for Δ*T* > 100 K, while being simultaneously within the uncertainty limit of the DLR measurement (Lab 3c reference principle) for the entire range of tested Δ*T* (Figure S11). Although TEM 3 showed a significant difference compared to other samples and the highest deviation from manufacturer specifications, almost all heat flow measurements (exception: data from Lab 6 at Δ*T* < 100 K) on this sample are within the uncertainty limit of the DLR measurement according to the reference principle (Lab 3b), as can be seen from Figure S11. Comparison of heat flow data from the absolute GHP-based measurement technique by DLR (Lab 3a) confirms that only measurement results by Lab 7 fall within the uncertainty limit of the GHP measurement. As mentioned earlier, the GHP-based measurement by DLR systematically overestimates the incident heat flow to the TEM due to heat losses if combined with an HFM block at the hot side of the measuring section. However, considering only DLR results obtained by the reference principle confirms that manufacturer specifications for ${\dot{Q}}_{\mathrm{in}}$ could be reproduced within the limits of uncertainty by measurements on TEM 1 only.

#### 3.4.3. Maximum Efficiency

**Figure 12:**
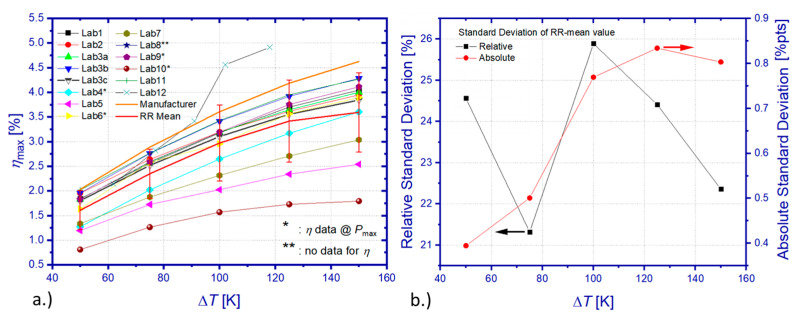
RR results for the maximum efficiency *η*_max_ in dependence on the temperature difference Δ*T* (**a**). The mean value (RR mean) and the standard deviation (shown by error bars for the RR mean) have been calculated from all available data sets. For comparison, manufacturer specifications are plotted. The absolute and relative standard deviations are shown in (**b**).

Power output and heat flow results have been used to determine the maximum efficiency of the tested TEM. As can be seen from Figure 12, the absolute standard deviation of the resulting mean value (RR mean), which was calculated from all data sets, ranges from 0.4%pts to 0.83%pts within the tested interval of Δ*T*. This corresponds to a relative standard deviation between 21.3% and 25.9% from the mean value. Comparison of the RR mean values with manufacturer specifications revealed a deviation between 17.6% and 22.3%.

**Figure 13:**
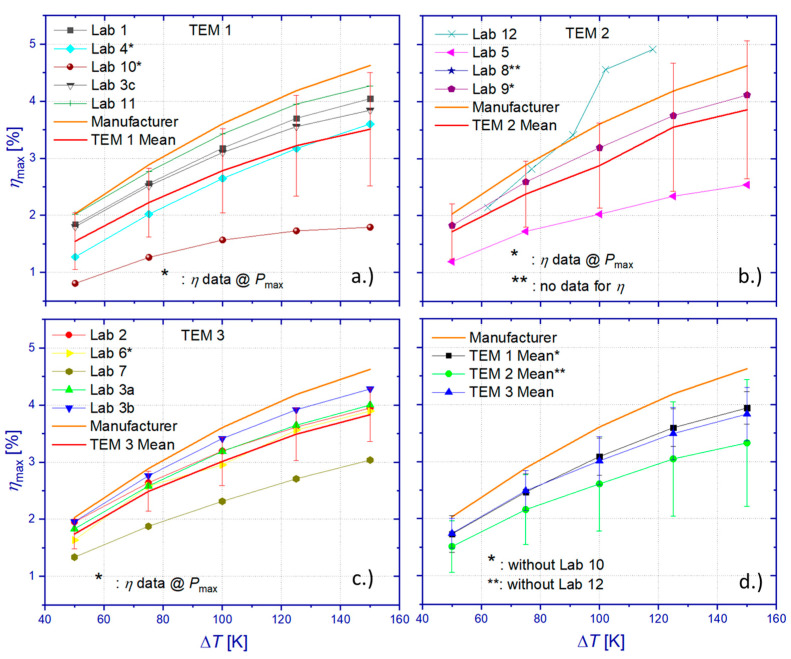
RR results for the maximum efficiency *η*_max_ versus temperature difference Δ*T* shown separately for TEM1 (**a**), TEM2 (**b**), and TEM3 (**c**). Mean values and standard deviation, displayed as error bars, have been calculated from all data sets of each TEM. Missing data sets or specification of efficiency at maximum power as well as manufacturer specifications are indicated. Mean values of the TEM are compared in (**d**).

Evaluation of all TEM-specific results of the maximum efficiency (Figure 13) leads to significant standard deviations of 0.5%pts–0.99%pts (TEM 1), 0.48%pts–1.2%pts (TEM 2), and 0.26%pts–0.46%pts (TEM 3). Omitting results from Lab 10 (TEM 1) and Lab 12 (TEM 2) yields reduced relative standard deviations of the TEM-specific mean values of 0.3%pts–0.28%pts (TEM 1) and 0.44%pts–1.1%pts (TEM 2), respectively. Even with the omission of data from Lab 10 and Lab 12, relative differences between TEM-specific mean values of *η*_max_ and manufacturer specifications equal 15.1% (TEM 1), 28.1% (TEM 2), and 17.1% (TEM 3).

Comparison between TEM-specific RR data on *η*_max_ and DLR results obtained by the reference principle for heat flow determination (Figure S12) shows that only a few laboratories (TEM 1: Lab 4 at Δ*T* < 100 K, TEM 3: Lab 7) obtained results outside the specified uncertainty budget by DLR. Accordance of the *η*_max_ results of the RR and DLR measurements, which are based on the reference principle, is recognized for data from Lab 1, 4, and 11 (TEM 1) and from Lab 2 and 6 (TEM 3). However, comparing participants’ data for *η*_max_ with DLR results obtained on the basis of heat flow results from the GHP method reveals that only data from Lab 2 and Lab 6 delivered consistent results within the determined uncertainty budget *u*(*η*_max_) of reference principle measurements by DLR. Any coincidence between DLR and RR results for *η*_max_ is caused most likely by compensation effects from over- or underestimating measurement results of the power output and the heat flow, which might partly originate from deviations of temperature conditions due to different stabilization criteria and different approaches for the compensation of the current-dependent Peltier effect on the temperature boundary conditions. The best accordance with DLR results was achieved for data from Lab 4, which kept within the uncertainty budgets of DLR for every tested module measurand.

## 4. Conclusions

Uncertainty analyses of DLR measurement techniques for the determination of the power output, the heat flow, and the TEM efficiency were presented. Analyses of standard deviations and sensitivity coefficients of individual input parameters to the measurement instruction for *P*_max_ revealed a combined uncertainty 0.27% < *u*(*P*_max_) < 0.54% (*k* = 2). The highest uncertainty contributions to power measurements originate from current measurements, which showed a strong correlation to the temperature difference over the TEM, Δ*T*. The uncertainty contribution of the current measurement showed maximum values close to the optimum operation conditions of maximum power output and maximum efficiency. The combined uncertainty of heat flow measurement by the reference principle revealed an almost temperature-independent value of *u*(${\dot{Q}}_{\mathrm{In}}$)~16% (*k* = 2). It is dominated by the uncertainty of the thermal conductivity of the HFM, followed by the contribution of the temperature gradient measurement, which is sensitive to thermal transients, signal noise, and any violation of the one-dimensionality of heat flow through the HFM.

The combined uncertainty of heat flow measurement by the GHP technique showed significantly lower uncertainties 0.27% < *u*(${\dot{Q}}_{\mathrm{In}}$) < 0.34% (*k* = 2), which stem from a parasitic heat exchange within the GHP system between the MH and its guard heaters. This heat exchange is triggered by the imperfect temperature homogeneity of the guard heater surfaces. Mainly, the heat flow from the MH leads to an inevitable temperature drop along the heat flow path from the MH to the coupling surface of the measuring section, which does not allow for the perfect thermal guarding of the MH by the guard heaters. However, despite lower uncertainties of the GHP-based measurement compared to the reference principle, DLR results showed a deviation between both methods, which scaled linearly from 5.7% and 8.7% of the nominal heat release by the MH within the tested interval of Δ*T*. This deviation corresponded to and originated from the lateral heat losses of the HFM, which was located in this study on the hot side of the measuring section between the GHP block and the TEM. Significantly smaller deviations of 1.5% [49] and 1.14% [51] have been observed between both methods during previous studies with a cold side HFM instead. Then, the HFM is operated at a lower mean temperature, which consequently reduces heat losses to the surroundings in the measurement compartment. Sensitivity coefficients and standard uncertainties for power output and heat flow were used for the calculation of individual uncertainty contributions of the module efficiency. The resulting combined uncertainty of the maximum module efficiency was specified for the reference principle at *u*(_Mmax_)~16% (*k* = 2) and for GHP-based measurements at 0.37% < *u*(*η*_max_) < 0.64% (*k* = 2).

The comparison of TEM characterization results from an international RR campaign with twelve participating laboratories revealed severe deviations of *P*_max_, ${\dot{Q}}_{\mathrm{In}}$, and *η*_max_, with maximum standard uncertainties of 27.2% (*P*_max_), 59.2% (${\dot{Q}}_{\mathrm{In}}$), and 25.9% (*η*_max_), respectively. These deviations might be partially caused by possible dissimilarities in the properties of the tested TEM samples. However, analyses of RR results on individual TEMs confirmed likewise substantial discrepancies and point to significant measurement uncertainties, major differences in applied measurement conditions (temperatures, pressure, stabilization criteria), or inaccurate evaluation procedures. A comparison between TEM-specific RR data sets and manufacturer specifications has shown deviations between 12.6% and 46.9%. Only a few laboratories obtained results within the uncertainty budgets of DLR measurements. A distinct analysis of systematic reasons was not feasible in the course of the RR due to the lack of detailed information on individual device construction, applied measurement protocols, and data processing.

It should be stated that this RR test was conducted up to a moderate hot side temperature of 200 °C only. More severe practical difficulties and larger uncertainties can be expected, particularly for measurements of the heat flow at higher temperatures. Against the background of the continuous development of high-temperature thermoelectric modules on the one hand and the outcome of the RR campaign on the other hand, two main conclusions can be drawn. There is a distinct need for standardization activities in order to increase the accuracy and confidence level of TEM measurements. High-temperature TEM reference samples need to be made available, which would allow for the efficient qualification of characterization methods by determination of their deviations and apparent uncertainty budgets.

## Figures and Tables

**Table 1:** Information on the module type selected for RR tests according to manufacturer specifications. The geometry data refer to the cross-section area, which is available for thermal coupling. The nominal hot and cold side temperatures *T*_H_ and *T*_C_ give the testing conditions of the specified module properties.

Place of Origin	Geometry [mm^3^]	Max. *T*_H_ [°C]	Nominal *T*_H_/*T*_C_ [°C]	*R*_i_ [Ω]	*P*_max_ [W]	*η*_max_ [%]
USA	40 × 40 × 3.5	230	230/50	3.46	7.95	4.97

**Table 2:** Distribution pattern of the RR campaign. The order of listed laboratories corresponds to the chronological order of conducted measurements on a particular TEM sample.

TEM 1	TEM 2	TEM 3
Lab 1	Lab 12	Lab 2
Lab 4	Lab 5	Lab 6
Lab 10	Lab 8	Lab 7
Lab 3c	Lab 9	Lab 3a/3b
Lab 11	-/-	-/-

**Table 3:** The average uncertainty *u*(*P*)_avg_ is calculated as a mean value from uncertainties of power measurements *u*(*P*) (*k* = 2) at individual current set points. The average uncertainty *u*(*P*)_avg_ is given by absolute and relative values in dependence on the applied Δ*T*.

**Δ*T* [K]**	50	75	100	125	150
***u*** **(*P*)_avg_ [mW]**	5.27	9.72	14.07	18.21	24.26
***u*** **(*P*)_avg_ [%]**	2.33	2.02	1.63	1.21	1.25

## Data Availability

The data presented in this study are available in the manuscript and Supplementary Information of the article.

## Supplementary Materials

The following supporting information can be downloaded at: https://www.mdpi.com/article/10.3390/ma15051627/s1, Figure S1: Relative standard deviation of the mean value of the maximum power output *σ*(*P*_Max_) calculated from approximated heating and cooling curves of respective laboratory data sets. *σ*(*P*_Max_) is plotted for each of the three RR samples individually in an own graph (a—TEM1, b—TEM2, c—TEM3). Every legend reflects the chronological order of the conducted measurements by respective laboratories, Figure S2: Relative standard deviation of the mean value of the incident heat flow *σ*(*Q*_In_) calculated from approximated heating and cooling curves of respective laboratory data sets. *σ*(*Q*_In_) is plotted for each of the three RR samples individually in an own graph (a—TEM1, b—TEM2, c—TEM3). Every legend reflects the chronological order of the conducted measurements by respective laboratories, Figure S3: Relative standard deviation of the mean value of the maximum efficiency *σ*(*η*_Max_) calculated from approximated heating and cooling curves of respective laboratory data sets. *σ*(*η*_Max_) is plotted for each of the three RR samples individually in an own graph (a—TEM1, b—TEM2, c—TEM3). Every legend reflects the chronological order of the conducted measurements by respective laboratories, Figure S4: Absolute (left axes) and relative uncertainty (right axes) of power measurement *u*(*P*) in dependence of electric current flow *I*. The shown uncertainties are displayed in relation to ∆*T* at open loop conditions (a. 50 K, b. 75 K, c. 100 K, d. 125 K, e. 150 K) and for a coverage factor *k* = 1, Figure S5: Uncertainty contributions (*k* = 1) of current (a) and voltage (b) measurements to *u*(*P*) in dependence of normalized current *I*. The shown contributions are grouped in relation to ∆*T* at open loop conditions, Figure S6: Uncertainty contributions (*k* = 1) to *u*(*I*) given by uncertainty of current measurement over the shunt resistor (a) and the uncertainty of the referenced value of the shunt resistor (b) in dependence of normalized current *I*. The shown contributions are grouped in relation to ∆*T* at open loop conditions, Figure S7: Repetitive measurement (*N* = 20) of temperature profiles by five thermocouples within an HFM under nominally stable temperature conditions (∆*T* = 116.9 K) close to conditions of the optimum current flow for maximum efficiency operation of TEM3 (a). Resulting mean temperatures *T*_Mean_ are transferred to a linear approximation (b) for determination of a best estimate of the temperature gradient ∇*T* and its standard uncertainty *u*(∇*T*), Figure S8: Uncertainty contributions (*k* = 1) to the combined uncertainty of heat flow determination according to the reference principle. The uncertainty of the thermal conductivity (a) of the employed heat flow meter contributes most compared to the uncertainty contributions of the temperature gradient measurement and the geometry of the heat flow meter (b), Figure S9: The effective thermal conductance *X*_Guard-GHP_ between the metering heater (MH) of the GHP and the guard heater system (a) was obtained from an imbalance experiment for a GHP temperature range between 373 K < *T*_MH_ < 773 K (black squares). The left figure shows a linear approximation of *X*_Guard-GHP_ up to 1023 K (blue circles). The maximum temperature difference along the heat transmission path from the MH to the exit point of the GHP at the begin of the measuring section (b) was determined experimentally during characterization of TEM3, Figure S10: The combined uncertainty of the maximum efficiency contains contributions from the power output measurement (a) and heat flow measurement (b). Both contributions have been calculated with measurement data obtained from the reference principle and the GHP-method. All uncertainty contributions are displayed for a coverage factor *k* = 1, Figure S11: RR results for incident heat flow *Q*_In_ at conditions of maximum efficiency operation are shown in dependence of the temperature difference ∆*T*. The results are shown separately for measurements on TEM1 (a—without Lab 10), and TEM3 (b). DLR results show uncertainties of heat flow measurement (*k* = 2) as error bars. Specifications of heat flow data from conditions of maximum power output instead of maximum efficiency operation are indicated within the figures, Figure S12: RR results for the maximum efficiency *η*_Max_ are shown in dependence of the temperature difference ∆*T*. The results are shown separately for measurements on TEM1 (a—without Lab 10), and TEM3 (b). DLR results show uncertainties of efficiency measurement (*k* = 2) as error bars. Specifications of underlying heat flow data from conditions of maximum power output instead of maximum efficiency operation are indicated within the figures, Table S1:Best estimates (mean values), sensitivity coefficients, and standard uncertainty contributions for determination of the combined uncertainty *u*(*P*) for power output measurement. The analysis is accomplished for every set value of electric current flow and for different ∆*T*, which are related to the temperature differences over the TEM at open loop conditions, Table S2: Best estimates (mean values), sensitivity coefficients, and standard uncertainty contributions for determination of the combined uncertainty *u*(*P*_Max_) for the measurement of maximum power output *P*_Max_. All values have been derived from parabolic approximations of raw data *P*(*I*,*t*) and averaged values *P*(*I*)_Mean_. The analysis is accomplished in dependency of ∆*T* and evaluated at optimum current flow for maximum power output. ∆*T*_PMax_ is indicated accordingly, Table S3: Best estimates (mean values), sensitivity coefficients, and standard uncertainty contributions for determination of the combined *uncertainty u(*${\dot{Q}}_{\mathrm{Ref}}$*)* of heat flow measurement according to the reference principle. The analysis is accomplished for conditions of maximum efficiency operation. All values are displayed in dependency of Δ*T*_ηMax_.
