# Supplementary material for: International Round Robin Test of Thermoelectric Generator Modules
Source: Materials (Basel). 2022 Feb 22;15(5):1627. doi: 10.3390/ma15051627 (PMC8911437; doi:10.3390/ma15051627)
Supplement: Supplementary file 1 [file materials-15-01627-s001.zip › materials-1593197-supplementary.pdf]

Supplementary Materials

International Round Robin Test of Thermoelectric Generator Modules

Pawel Ziolkowski [1,*], Przemyslaw Blaschkewitz [1], Byungki Ryu [2], SuDong Park [2] and Eckhard Müller [1,3]

1 German Aerospace Center (DLR)—Institute of Materials Research, Linder Hoehe, D-51147 Cologne, Germany; przemyslaw.blaschkewitz@dlr.de (P.B.); eckhard.mueller@dlr.de (E.M.)
2 Energy Conversion Research Center, Electrical Materials Research Division, Korea Electrotechnology Research Institute (KERI), Changwon 51543, Korea; byungkiryu@keri.re.kr (B.R.); john@keri.re.kr (S.P.)
3 Institute of Inorganic and Analytical Chemistry, Justus Liebig University Gießen, Heinrich-Buff-Ring 17, D-35392 Gießen, Germany
* Correspondence: pawel.ziolkowski@dlr.de

S1. Standard deviation of mean values of approximated data sets from heating and cooling sequences

The comparison of the RR data is based on mean values, which were calculated for each property from approximated result functions obtained during the heating and cooling sequence of the conducted temperature cycle. Standard deviations between approximated data sets for heating and cooling sequences have not been determined for measurements of laboratories 8 and 12 on TEMs, since these participants delivered only results from the heating sequence.

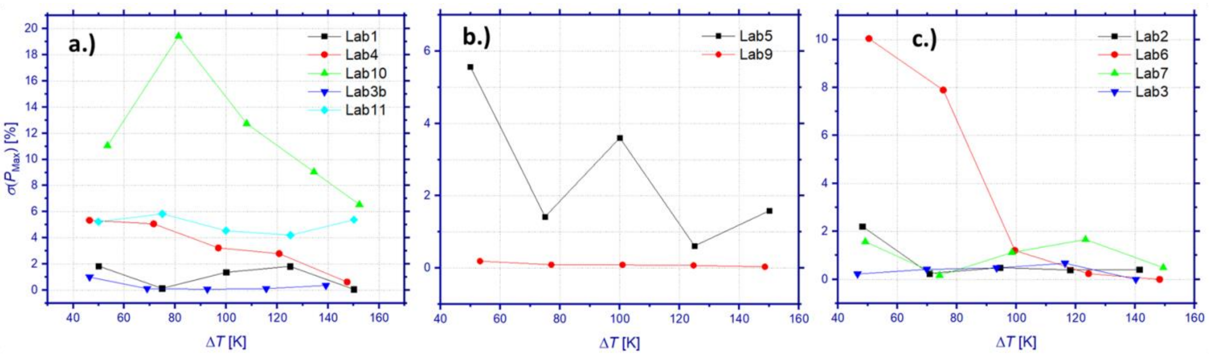

Figure S1. Relative standard deviation of the mean value of the maximum power output $\sigma(P_{Max})$ calculated from approximated heating and cooling curves of respective laboratory data sets. $\sigma(P_{Max})$ is plotted for each of the three RR samples individually in an own graph (a – TEM1, b – TEM2, c – TEM3). Every legend reflects the chronological order of the conducted measurements by respective laboratories.

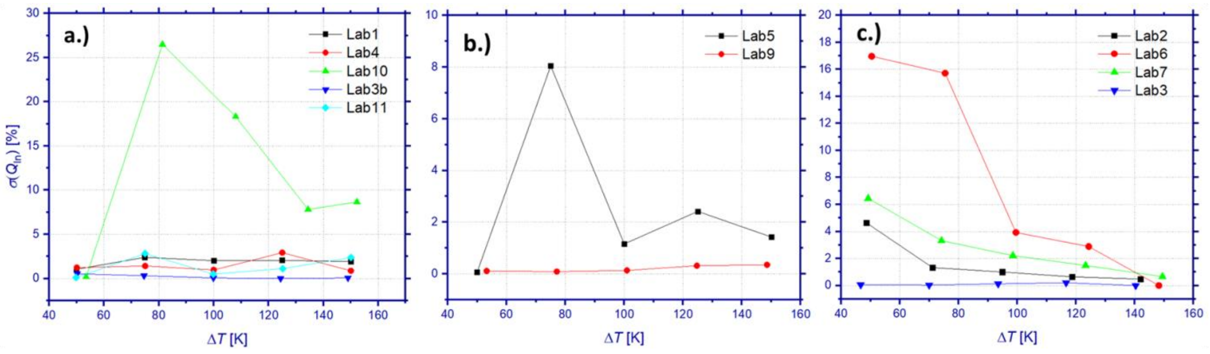

Figure S2. Relative standard deviation of the mean value of the incident heat flow $\sigma(Q_{In})$ calculated from approximated heating and cooling curves of respective laboratory data sets. $\sigma(Q_{In})$ is plotted for each of the three RR samples individually in an own graph (a – TEM1, b – TEM2, c – TEM3). Every legend reflects the chronological order of the conducted measurements by respective laboratories.

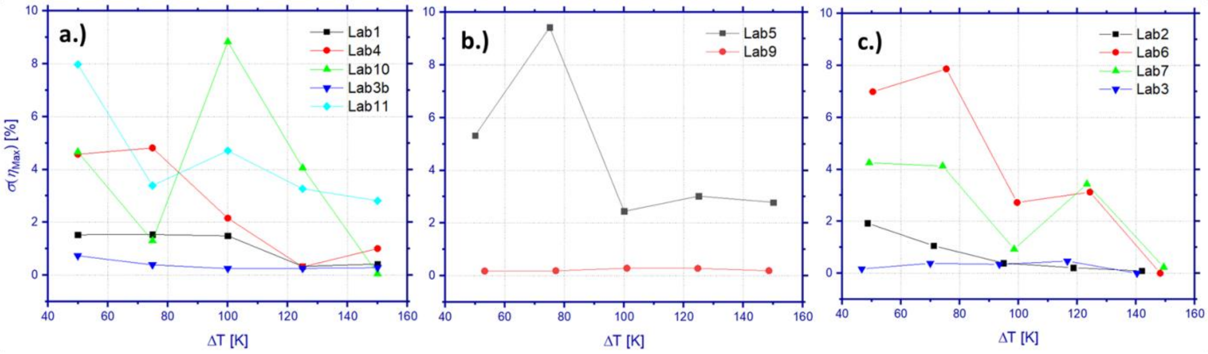

Figure S3. Relative standard deviation of the mean value of the maximum efficiency $\sigma(\eta_{Max})$ calculated from approximated heating and cooling curves of respective laboratory data sets. $\sigma(\eta_{Max})$ is plotted for each of the three RR samples individually in an own graph (a – TEM1, b – TEM2, c – TEM3). Every legend reflects the chronological order of the conducted measurements by respective laboratories.

S2. Uncertainty of power measurement - Lab 3a/3b - sample TEM3

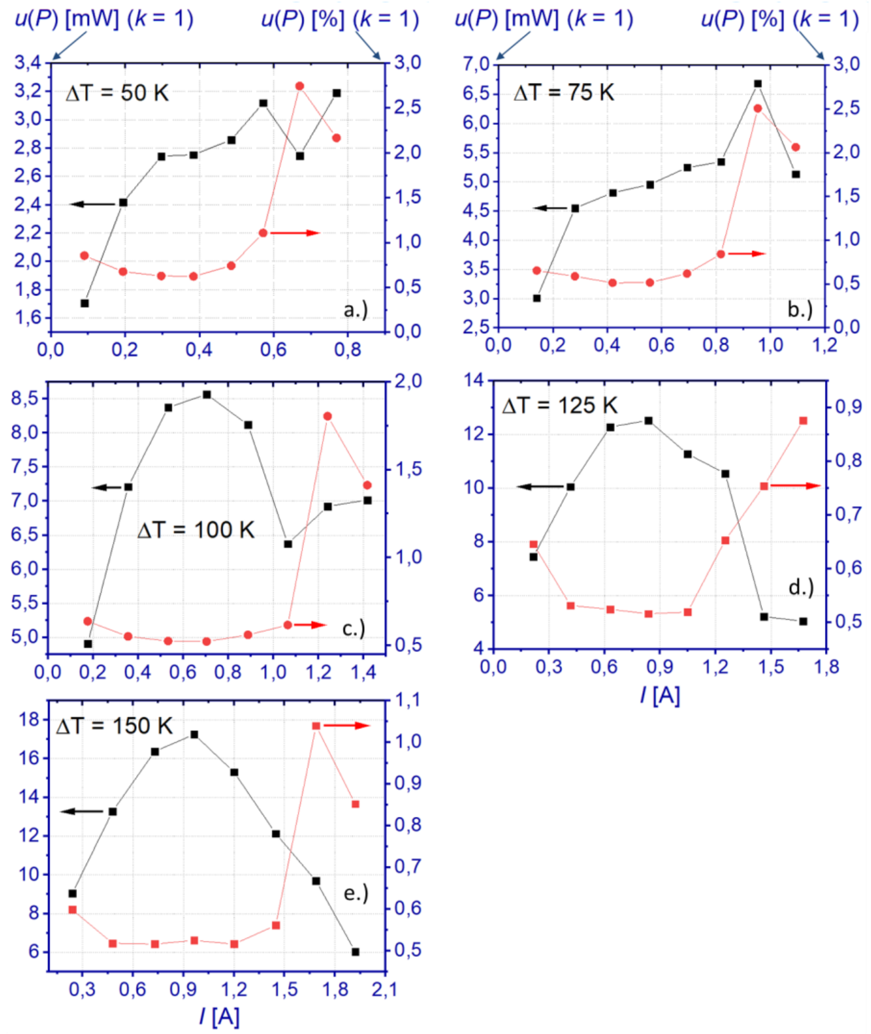

Figure S4. Absolute (left axes) and relative uncertainty (right axes) of power measurement $u(P)$ in dependence of electric current flow I. The shown uncertainties are displayed in relation to ΔT at open loop conditions (a. 50 K, b. 75 K, c. 100 K, d. 125 K, e. 150 K) and for a coverage factor $k = 1$.

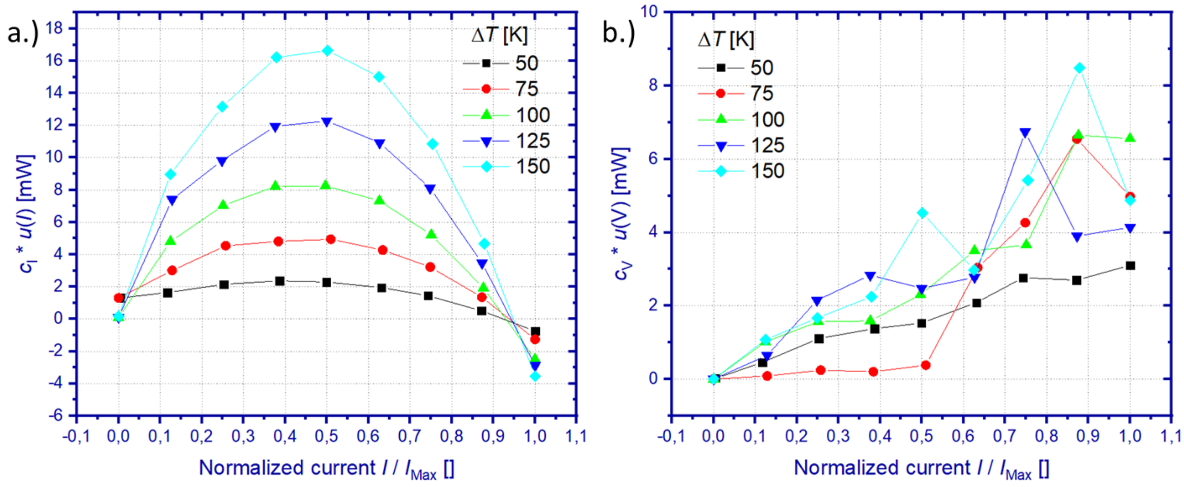

Figure S5. Uncertainty contributions (k = 1) of current (a) and voltage (b) measurements to $u(P)$ in dependence of normalized current I. The shown contributions are grouped in relation to ΔT at open loop conditions.

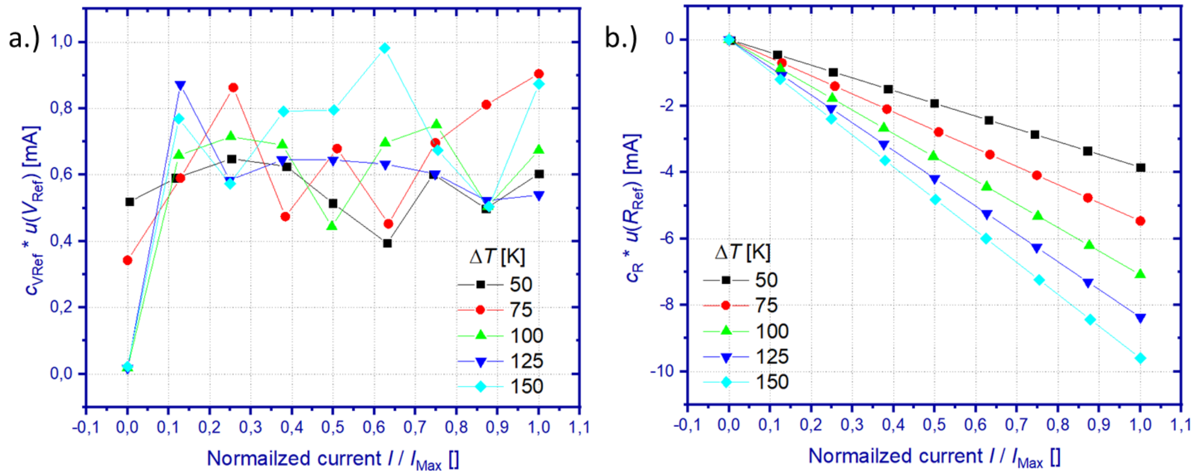

Figure S6. Uncertainty contributions (k = 1) to $u(I)$ given by uncertainty of current measurement over the shunt resistor (a) and the uncertainty of the referenced value of the shunt resistor (b) in dependence of normalized current I. The shown contributions are grouped in relation to ΔT at open loop conditions.

Table S1. Best estimates (mean values), sensitivity coefficients, and standard uncertainty contributions for determination of the combined uncertainty $u(P)$ for power output measurement. The analysis is accomplished for every set value of electric current flow and for different ΔT, which are related to the temperature differences over the TEM at open loop conditions.

ΔT = 50 K								
$c_V = I$ [mA]	90.3	194.3	297.3	384	485.5	571.7	670	769.1
$c_I = V$ [V]	2.21	1.83	1.47	1.15	0.79	0.49	0.14	-0.19
$u(I)$ [mA]	0.743	1.17	1.61	1.99	2.46	2.92	3.39	3.89
$u(V)$ [mV]	5.06	5.7	4.63	3.98	4.3	4.84	4.03	4.03
P [mW]	199.8	357.4	437.1	442.3	385.5	281.7	100.1	-147.3
$u(P)$ [mW]	1.7	2.41	2.74	2.75	2.85	3.11	2.74	3.19
c_{VRef} [1/Ω]	10							
c_R [A/Ω]	-0.9	-1.94	-2.97	-3.84	-4.85	-5.71	-6.7	-7.69
V_{Ref} [mV]	9.03	19.43	29.73	38.4	48.55	57.17	67	76.91
$u(V_{Ref})$ [μV]	59.02	64.84	62.52	51.46	39.35	60.16	49.81	60.37
$u(R_{Ref})$ [mΩ]	0.5							
ΔT = 75 K								
$c_V = I$ [mA]	140.7	281.1	419.4	556.9	693.3	818	953.6	1093
$c_I = V$ [V]	3.27	2.75	2.23	1.72	1.22	0.77	0.28	-0.227
$u(I)$ [mA]	0.918	1.65	2.15	2.87	3.5	4.15	4.84	5.54
$u(V)$ [mV]	0.59	0.863	0.474	0.678	4.38	5.21	6.87	4.55
P [mW]	461	774.4	937.9	960	847.8	635	266.8	-248.9

$u(P)$ [mW]	3.01	4.54	4.81	4.95	5.24	5.34	6.68	5.13
c_{VRef} [1/Ω]	10							
c_R [A/Ω]	-1.4	-2.81	-4.19	-5.56	-6.93	-8.18	-9.53	-10.93
V_{Ref} [mV]	14.07	28.11	41.94	55.69	69.33	81.8	95.36	109.3
$u(V_{Ref})$ [μV]	59	86.3	47.42	67.89	45.24	69.61	81.12	90.45
$u(R_{Ref})$ [mΩ]	0.5							
**ΔT = 100 K**								
**$c_V = I$ [mA]**	**176.5**	**355.6**	**533.3**	**704**	**887.6**	**1064**	**1241**	**1417**
$c_I = V$ [V]	4.36	3.67	2.98	2.32	1.63	0.97	0.31	-0.35
$u(I)$ [mA]	1.1	1.92	2.75	3.55	4.49	5.37	6.23	7,12
$u(V)$ [mV]	5.75	4.4	2.97	3.27	3.94	3.44	5.36	4.63
P [mW]	769.5	1306	1592	1637	1447	1033	383.5	-496.8
$u(P)$ [mW]	4.9	7.21	8.37	8.56	8.11	6.37	6.92	7.01
c_{VRef} [1/Ω]	10							
c_R [A/Ω]	-1.76	-3.55	-5.33	-7.04	-8.87	-10.64	-12.41	-14.17
V_{Ref} [mV]	1.76	3.55	5.33	7.04	8.87	10.64	12.41	14.17
$u(V_{Ref})$ [μV]	65.87	71.45	69	44.37	69.63	75.07	50.45	67.42
$u(R_{Ref})$ [mΩ]	0.5							
**ΔT = 125 K**								

$c_V = I$ [mA]	215.1	415.6	629.5	836.6	1049	1252	1461	1674
$c_I = V$ [V]	5.35	4.55	3.71	2.9	2.06	1.28	0.47	-0.34
$u(I)$ [mA]	1.38	2.16	3.21	4.23	5.28	6.29	7.32	8.39
$u(V)$ [mV]	3	5.17	4.5	2.96	2.64	5.39	2.67	2.47
P [mW]	1152	1891	2341	2427	2171	1615	692.1	-575.7
$u(P)$ [mW]	7.44	10.05	12.27	12.52	11.27	10.54	5.21	5.04
c_{VRef} [1/Ω]	10							
c_R [A/Ω]	-2.15	-4.15	-6.29	-8.36	-10.49	-12.52	-14.61	-16.74
V_{Ref} [mV]	21.51	41.56	62.95	83.66	104.9	125.2	146.1	167.4
$u(V_{Ref})$ [μV]	87.21	58.37	64.51	64.46	63.24	60.3	52.22	53.93
$u(R_{Ref})$ [mΩ]	0.5							
ΔT = 150 K								
$c_V = I$ [mA]	239.2	477.9	728.2	963.2	1200	1448	1687	1920
$c_I = V$ [V]	6.31	5.35	4.35	3.41	2.47	1.49	0.55	0.37
$u(I)$ [mA]	1.42	2.46	3.73	4.88	6.08	7.27	8.45	9.64
$u(V)$ [mV]	4.48	3.49	3.08	4.7	2.48	3.74	5.03	2.54
P [mW]	1510	2560	3170	3285	2964	2161	931.9	-708.1
$u(P)$ [mW]	9.04	13.27	16.37	17.25	15.3	12.13	9.68	6.03
c_{VRef} [1/Ω]	10							

c_R [A/Ω]	-2.39	-4.77	-7.28	-9.63	-12	-14.48	-.16.87	-19.2
V_{Ref} [mV]	23.92	47.79	72.82	96.32	120	144.8	168.7	192
$u(V_{Ref})$ [µV]	76.91	57.33	79.13	79.55	98.16	67.42	50.45	87.38
$u(R_{Ref})$ [mΩ]	0.5							

Table S2. Best estimates (mean values), sensitivity coefficients, and standard uncertainty contributions for determination of the combined uncertainty $u(P_{Max})$ for the measurement of maximum power output P_{Max}. All values have been derived from parabolic approximations of raw data $P(I,t)$ and averaged values $P(I)_{Mean}$. The analysis is accomplished in dependency of ΔT and evaluated at optimum current flow for maximum power output. ΔT_{PMax} is indicated accordingly.

ΔT_{PMax} [K]	46.44		69.79		93.38		116.8		140.2	
Input data set	P	P_{Mean}	P	P_{Mean}	P	P_{Mean}	P	P_{Mean}	P	P_{Mean}
$\|a\|$ [Ω]	3.51	3.49	3.64	3.64	3.76	3.75	3.87	3.87	3.94	3.95
$u(a)$ [mΩ]	3.65	16.86	3.31	10.49	3.44	11.89	2.34	8.95	2.15	6.89
b [V]	2.5	2.48	3.75	3.75	4.97	4.96	6.13	6.13	7.2	7.21
$u(b)$ [mV]	2.91	13.45	3.75	11.91	5.06	17.51	4.07	15.57	4.29	13.77
c [mW]	1.34	2.74	3.25	3.37	6.39	7.12	7.31	6.59	8.31	7.45
$u(c)$ [mW]	0.48	2.22	0.88	2.81	1.54	5.32	1.46	5.6	1.77	5.67
c_a [W/Ω]	0.127	0.126	0.264	0.264	0.436	0.436	0.626	0.626	0.833	0.832
c_b [A]	0.356	0.356	0.514	0.514	0.66	0.66	0.791	0.791	0.912	0.912
c_c []	1									
$c_a*u(a)$ [mW]	0.463	2.14	0.876	2.78	1.5	5.19	1.47	5.61	1.79	5.74
$c_b * u(b)$ [mW]	1.04	4.79	1.93	6.13	3.34	11.57	3.23	12.33	3.91	12.56

c_c * u(c) [mW]	0.481	2.22	0.884	2.81	1.54	5.32	1.46	5.6	1.77	5.67
$I_{Opt,P}$ [mA]	356.4	356.1	514.7	514.6	660.3	660.8	791.7	791.6	912.6	912.6
P_{Max} [W]	0.447	0.446	0.969	0.968	1.648	1.647	2.435	2.435	3.297	3.298
$u(P_{Max})$ [mW] (k = 1)	1.234	5.697	2.298	7.295	3.97	13.755	3.835	14.652	4.652	14.93
$u(P_{Max})$ [%] (k = 1)	0.275	1.277	0.237	0.752	0.24	0.834	0.157	0.601	0.141	0.452

S3. Uncertainty of heat flow measurement - Lab 3a/3b - sample TEM3

The uncertainty of the heat flow measurement by the reference principle $u(\dot{Q}_{Ref})$ is given by Equation (S1). It contains a geometric sum of individual uncertainty contributions from every input variable X of the measurement function (Equation (7) in article). Every contribution consists of a product of the respective standard uncertainty $u(X)$ and the corresponding sensitivity coefficient c_X. Sensitivity coefficients equal partial derivatives of the measurement function with respect to a respective input variable. Best estimates (mean values) of every input variable are taken for the evaluation of the sensitivity coefficients.

$$u(\dot{Q}_{REF}) = \sqrt{c_A^2 u(A_M)^2 + c_\kappa^2 u(\kappa_M)^2 + c_{\nabla T}^2 u(|\nabla T_M|)^2} \quad \textbf{(S1)}$$

$$c_A = \frac{d\dot{Q}_{REF}}{dA_M} = \kappa_M \cdot |\nabla T_M| \quad \textbf{(S2)}$$

$$c_\kappa = \frac{d\dot{Q}_{REF}}{d\kappa} = A_M \cdot |\nabla T_M| \quad \textbf{(S3)}$$

$$c_{\nabla T} = \frac{d\dot{Q}_{REF}}{d\nabla T} = A_M \cdot \kappa_M \quad \textbf{(S4)}$$

Standard uncertainties and best estimates of input variables can be determined from experimental results or specifications of calibration certificates. In this study a heat flow meter (HFM) has been used at the hot side of the tested TEMs, which was manufactured from Ni with dimensions of 40 x 40 x 60 mm^3. The thermal conductivity of the HFM material κ_M was investigated by a laser flash apparatus (LFA 427, Netzsch Gerätebau GmbH) for determination of the thermal diffusivity and by a differential scanning calorimeter (DSC 404, Netzsch Gerätebau GmbH) for measurement of the specific heat. According to calibration certificates of both instruments the relative standard uncertainty of the resulting thermal conductivity equals $u(\kappa_M)$ = ±8% (k = 1). The standard uncertainty of the cross section of the HFM $u(A_M)$ is expressed in a sub-model under consideration of the manufacturing tolerance of the HFM. The cross-section $A_M = W \cdot L$ equals the product of the width (W) and the length (L) of the HFM (dimensions perpendicular to the direction of heat flow). This uncertainty contribution combines best estimates for W and L (each 40 mm) and their standard uncertainties $u(W)$ and $u(L)$, which correspond to the manufacturing tolerance of ± 0.1 mm.

$$u(A_M) = \sqrt{c_W^2 u(W)^2 + c_L^2 u(L)^2} \quad \textbf{(S5)}$$

$$c_W = \frac{dA_M}{dW} = L \quad \textbf{(S6)}$$

$$c_Y = \frac{dA_M}{dL} = W \quad \textbf{(S7)}$$

Best estimates and standard uncertainties of the temperature gradient ∇T_M have been derived from an evaluation of temperature profiles, which were measured within the HFM by means of five type-N thermocouples (0.5 mm diameter, Inconel sheath, 1NI05/1000/MP/FM.N, ThermoExpert). After thermal stabilization of the measuring section temperatures have been measured 20 times at every sensor location. Figure S7a shows an exemplary set of temperature

data and the calculated mean values T_{Mean} in dependence of the sensor location. The standard deviation of T_{Mean} is indicated by error bars and shows maximum values of less than 0.7 K. T_{Mean} is forwarded as a function of the sensor location to a linear approximation (Figure S7b) for the determination of the best estimate ∇T and its standard uncertainty $u(\nabla T)$.

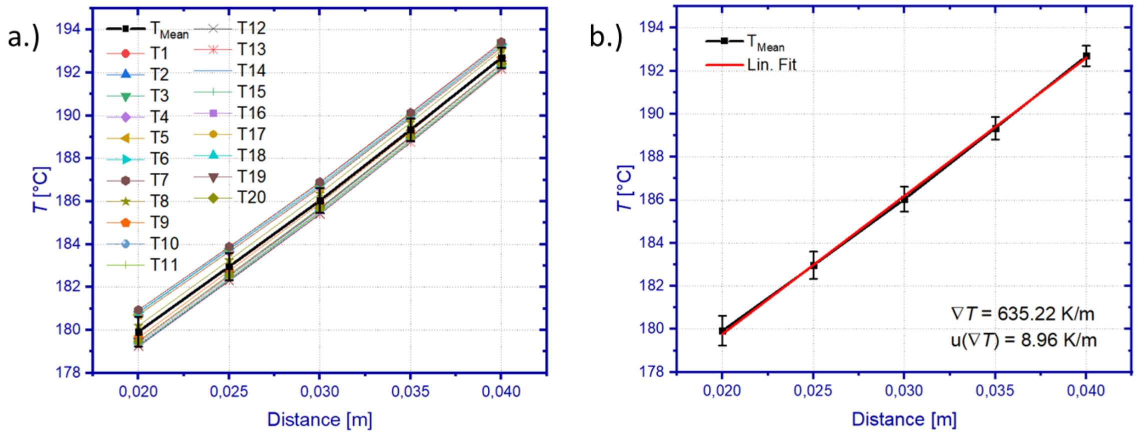

Figure S7. Repetitive measurement (N = 20) of temperature profiles by five thermocouples within an HFM under nominally stable temperature conditions (ΔT = 116.9 K) close to conditions of the optimum current flow for maximum efficiency operation of TEM3 (a). Resulting mean temperatures T_{Mean} are transferred to a linear approximation (b) for determination of a best estimate of the temperature gradient ∇T and its standard uncertainty $u(\nabla T)$.

The following figure summarizes individual uncertainty contributions to the combined uncertainty for heat flow determination according to the reference principle. The shown values relate to heat flow results obtained for conditions close to maximum efficiency operation.

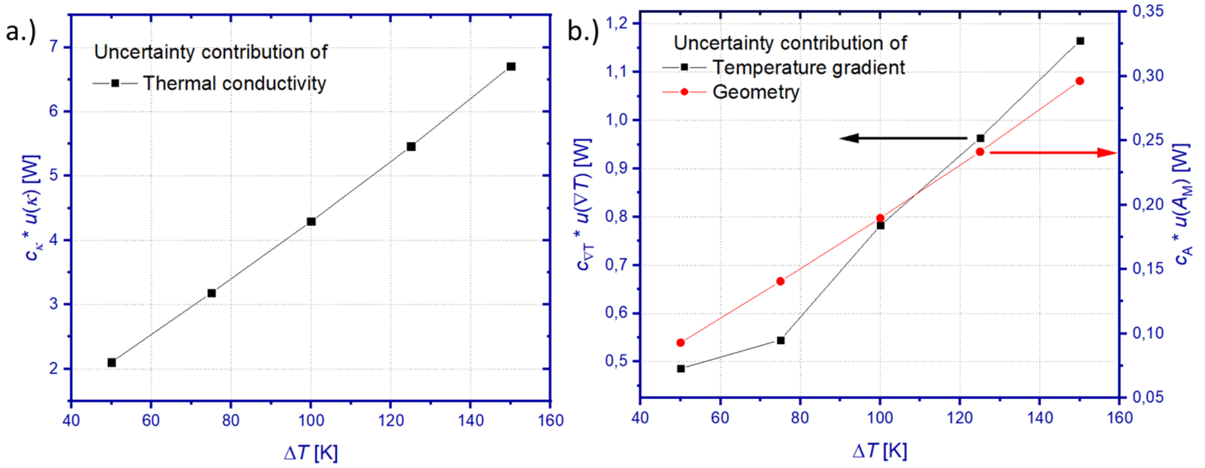

Figure S8. Uncertainty contributions (k = 1) to the combined uncertainty of heat flow determination according to the reference principle. The uncertainty of the thermal conductivity (a) of the employed heat flow meter contributes most compared to the uncertainty contributions of the temperature gradient measurement and the geometry of the heat flow meter (b). .

The following table summarizes underlying input data for the expression of the combined uncertainty $u(\dot{Q}_{Ref})$. The presented evaluation is given in dependence of the temperature difference at optimum current flow for maximum efficiency operation of TEM 3.

Table S3. Best estimates (mean values), sensitivity coefficients, and standard uncertainty contributions for determination of the combined *uncertainty* $u(\dot{Q}_{Ref})$ of heat flow measurement according to the reference principle. The analysis is accomplished for conditions of maximum efficiency operation. All values are displayed in dependency of $\Delta T_{\eta Max}$.

$\Delta T_{\eta Max}$ [K]	46.49	69.87	93.47	116.9	140.3
c_A [W/m²]	16431.65	24845.92	33528.41	42650.02	52369.82
$u(A_M)$ [m²]	$5.67 \cdot 10^{-6}$				
c_κ [m·K]	0.361	0.561	0.778	1.016	1.281
$u(\kappa)$ [W/m·K]	5.82	5.664	5.515	5.371	5.234
$c_{\nabla T}$ [m·W/K]	0.116	0.113	0.11	0.107	0.104
$u(\nabla T)$ [K/m]	4.174	4.811	7.089	8.968	11.129
$c_A * u(A_M)$ [W]	0.093	0.14	0.189	0.241	0.296
$c_\kappa * u(\kappa)$ [W]	2.103	3.18	4.291	5.459	6.703
$c_{\nabla T} * u(\nabla T)$ [W]	0.486	0.545	0.782	0.963	1.165
Q_{Ref} [W]	26.291	39.753	53.645	68.24	83.791
$u(Q_{Ref})$ [mW] (k = 1)	2.2	3.29	4.45	5.66	6.94
$u(Q_{Ref})$ [%] (k = 1)	8.38	8.29	8.30	8.29	8.28

The uncertainty $u(\dot{Q}_{GHP})$ of the guarded hot plate (GHP) measurement of Q_{GHP} is determined by the thermal cross-talk between the metering heater (MH) and the guard heater system, which is described in literature as the gap imbalance error [1]. This error is caused by the temperature difference between the guard heaters and the MH and introduces a parasitic heat exchange. Since only a finite number of sensor locations can be used for temperature imbalance detection, even a temperature difference between opposing sides of heaters (across their gaps), which is reduced to zero, will not necessarily ensure a vanishing net heat flow. Consequently, the systematic error component is influenced by the sensor positioning and the signal to noise ratio within the sensor circuits, while short-term fluctuations of the temperature control introduce an additional random component to the gap imbalance error. The thermal cross-talk due to this gap imbalance error is expressed by the parasitic heat exchange $Q_{Guard\text{-}GHP}$, which was studied in a previous work [2] by means of an intentional detuning of temperatures of the MH and the guard heaters. Concurrent measurements of the power consumption of all heaters under detuned temperature conditions allowed for a temperature dependent quantification of the effective thermal conductance $X_{Guard\text{-}GHP}$ between the guard heater system and the metering heater.

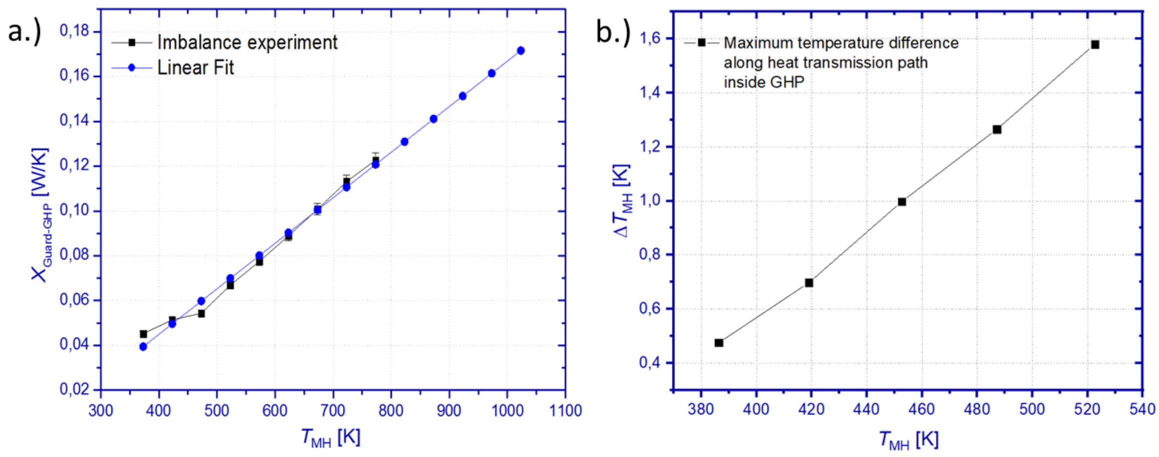

Figure S9. The effective thermal conductance $X_{Guard-GHP}$ between the metering heater (MH) of the GHP and the guard heater system (a) was obtained from an imbalance experiment for a GHP temperature range between 373 K < T_{MH} < 773 K (black squares). The left figure shows a linear approximation of $X_{Guard-GHP}$ up to 1023 K (blue circles). The maximum temperature difference along the heat transmission path from the MH to the exit point of the GHP at the begin of the measuring section (b) was determined experimentally during characterization of TEM3.

The heat flow generated by the MH introduces a temperature difference $\Delta T_{MH} = T_{MH} - T_{HC}$ between the MH (T_{MH}) and the heat flow exit point of the GHP at the hot coupling side of the measuring section which has the temperature T_{HC}. This leads to an inevitable thermal imbalance between the heat flow path from the MH to the measuring section and the guard heater system. ΔT_{MH} drops at a Cu-shell, which is located between the MH and the coupling surface of the GHP. Measurements of the maximum occurring temperature differences within the GHP-system ΔT_{HM} have been conducted in this study for the quantification of $u(\dot{Q}_{GHP})$ according to the following equation.

$$u(\dot{Q}_{GHP}) = \dot{Q}_{Guard-GHP} = X_{Guard-GHP} \cdot \Delta T_{MH} \quad \text{(S8)}$$

S4. Uncertainty of efficiency measurement - Lab 3a/3b - sample TEM3

The following figure summarizes uncertainty contributions of the power output measurement and the heat flow measurements to the combined uncertainty of the maximum TEM efficiency. The evaluation has been conducted with input data obtained from conditions of maximum efficiency operation.

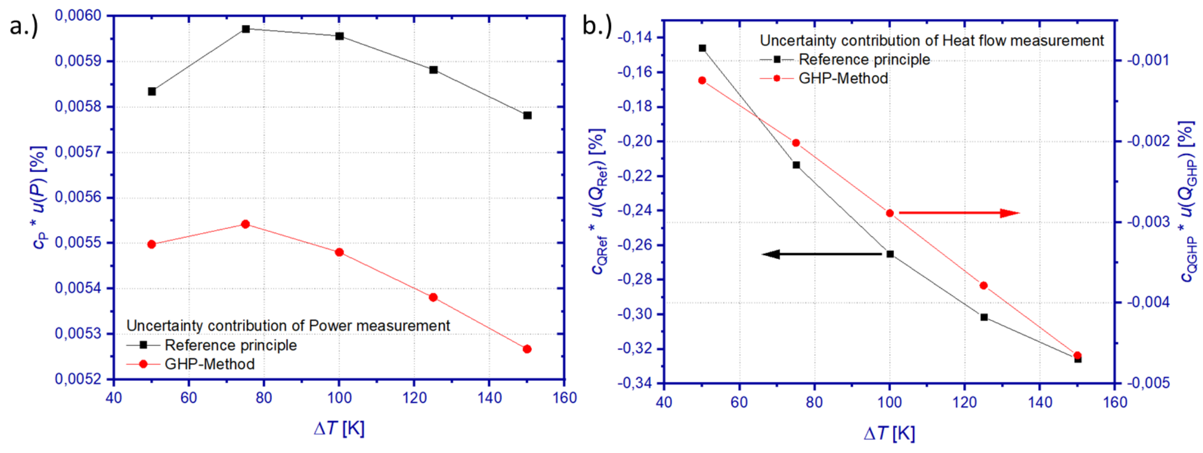

Figure S10. The combined uncertainty of the maximum efficiency contains contributions from the power output measurement (a) and heat flow measurement (b). Both contributions have been calculated with measurement data obtained from the reference principle and the GHP-method. All uncertainty contributions are displayed for a coverage factor k = 1.

S5. Heat flow and efficiency results for TEM 1 and TEM 3 with indication of DLR uncertainties

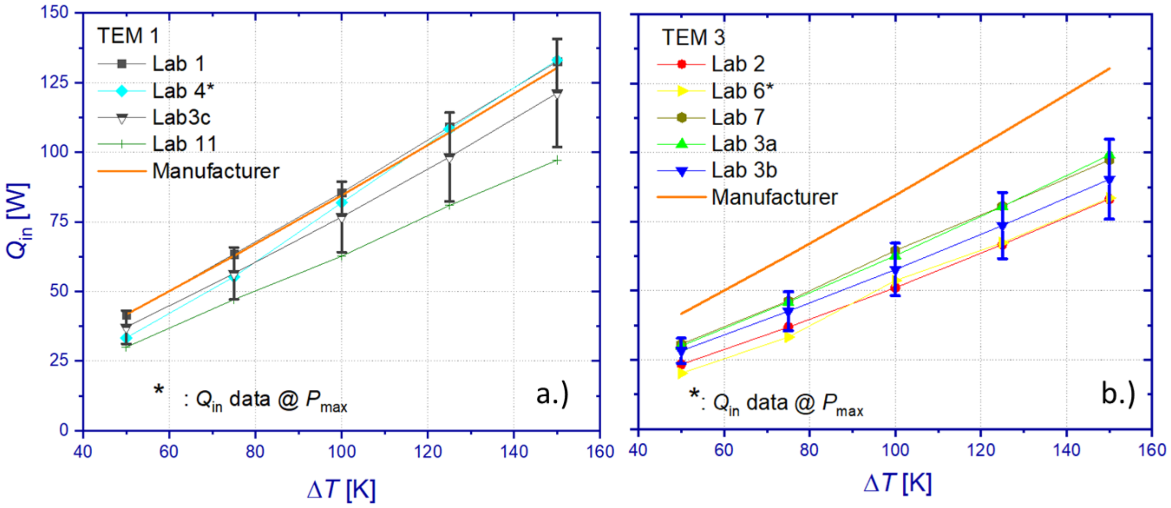

Figure S11. RR results for incident heat flow Q_{In} at conditions of maximum efficiency operation are shown in dependence of the temperature difference ΔT. The results are shown separately for measurements on TEM1 (a – without Lab 10), and TEM3 (b). DLR results show uncertainties of heat flow measurement (k = 2) as error bars. Specifications of heat flow data from conditions of maximum power output instead of maximum efficiency operation are indicated within the figures.

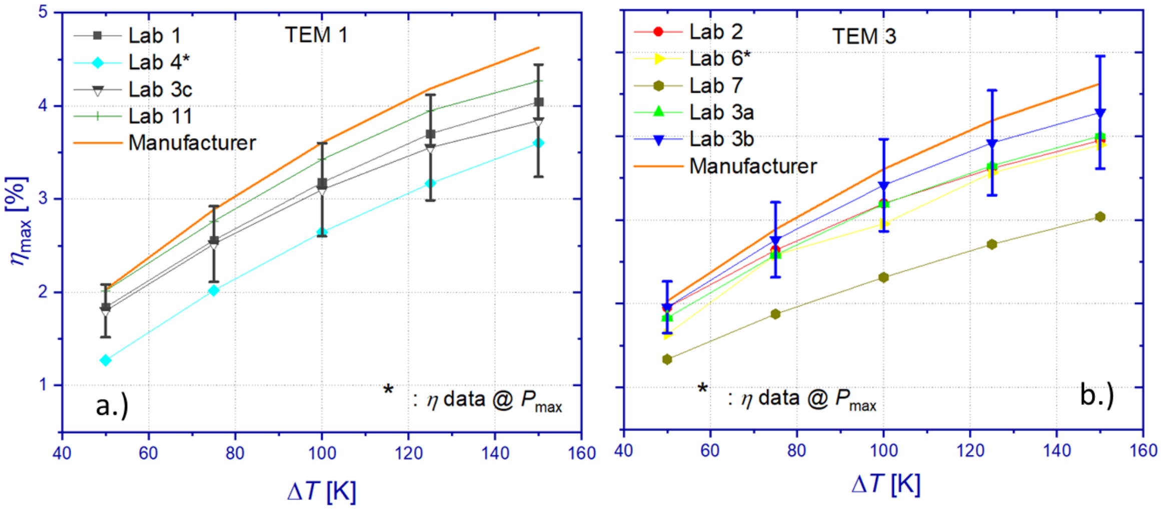

Figure S12. RR results for the maximum efficiency η_{Max} are shown in dependence of the temperature difference ΔT. The results are shown separately for measurements on TEM1 (a – without Lab 10), and TEM3 (b). DLR results show uncertainties of efficiency measurement (k = 2) as error bars. Specifications of underlying heat flow data from conditions of maximum power output instead of maximum efficiency operation are indicated within the figures.

References

1. *ASTM C177-19.* Standard Test Method for Steady-State Heat Flux Measurements and Thermal Transmission Properties by Means of the Guarded-Hot-Plate Apparatus, ASTM International: West Conshohocken, PA, USA, 2019.

2. Ziolkowski, P.; Blaschkewitz, P.; Müller, E. Heat Flow Measurement as a key to Standardization of Thermoelectric Generator Module Metrology: A Comparison of reference and absolute Techniques. *Measurement,* 2021, 167, 108273. https://doi.org/10.1016/j.measurement.2020.108273.
